# Reaction Trajectory Revealed by a Joint Analysis of Protein Data Bank

**DOI:** 10.1371/journal.pone.0077141

**Published:** 2013-11-11

**Authors:** Zhong Ren

**Affiliations:** 1 Center for Advanced Radiation Sources, The University of Chicago, Argonne, Illinois, United States of America; 2 Renz Research, Inc., Westmont, Illinois, United States of America; University of South Florida College of Medicine, United States of America

## Abstract

Structural motions along a reaction pathway hold the secret about how a biological macromolecule functions. If each static structure were considered as a snapshot of the protein molecule in action, a large collection of structures would constitute a multidimensional conformational space of an enormous size. Here I present a joint analysis of hundreds of known structures of human hemoglobin in the Protein Data Bank. By applying singular value decomposition to distance matrices of these structures, I demonstrate that this large collection of structural snapshots, derived under a wide range of experimental conditions, arrange orderly along a reaction pathway. The structural motions along this extensive trajectory, including several helical transformations, arrive at a reverse engineered mechanism of the cooperative machinery (Ren, companion article), and shed light on pathological properties of the abnormal homotetrameric hemoglobins from α-thalassemia. This method of meta-analysis provides a general approach to structural dynamics based on static protein structures in this post genomics era.

## Introduction

Protein crystallography, a powerful yet largely static technique, has greatly advanced our knowledge of protein structures by providing observation of atomic details, but at a cost of crystallization. Extending its capability to study functional dynamics has motivated decades of efforts, and remains to be a major challenge in structural biology today. The paradox is that a greater atomic detail, or higher resolution in crystallographic terms, demands more stringent lattice periodicity that hinders large-scale motions often required for function [1]. On the other hand, rapid progresses in structural genomics and broad applications of protein crystallography are contributing to the mounting entries of data in the Protein Data Bank (PDB) at a current growth rate greater than 10% annually [2]. Each entry represents a snapshot of a protein structure under a specific condition. The key question is whether a large amount of snapshots of a same protein would exhibit a well-ordered reaction pathway or merely a heap of different structures. If a sufficiently large and diverse collection of static structures is available, a joint analysis approach would reveal functional motions and lead to mechanistic understanding of a macromolecular system [3]. This meta-analysis [4] presented here in structural bioinformatics extends the commonly used pairwise structural comparison to a simultaneous evaluation of a large structural collection.

**Figure 1:**
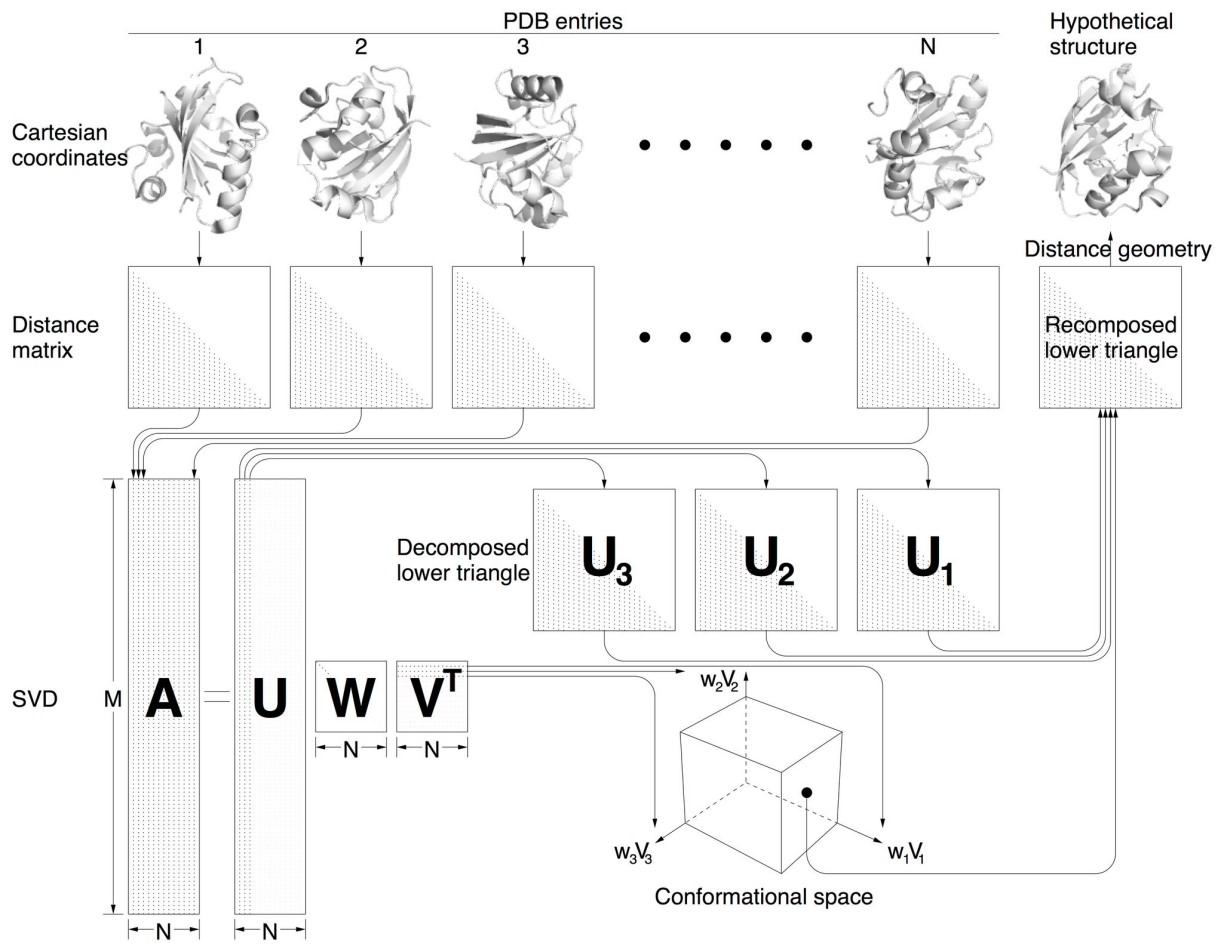
A flowchart of meta-analysis loop. Distance matrices are calculated from *N* related PDB entries in form of Cartesian coordinates. The lower triangles of the distance matrices are assembled into columns of matrix **A**. The first a few significant left singular vectors in **U** resulting from SVD are reconstructed to form decomposed lower triangles. The corresponding singular values and right singular vectors are presented in a multidimensional conformational space. Any given location in this space leads to a recomposed lower triangle by a linear combination of the significant lower triangles. Finally, a hypothetical structure is determined by distance geometry using all constraints in the recomposed lower triangle.

A flowchart (Figure 1) summarizes key steps in the analytical loop developed here. The companion article [5] provides a brief introduction to the structure and function relationship of human hemoglobin (Hb), and describes a reverse engineering approach to a mechanical model that depicts the inner workings of a cooperative machinery based on evidences collected from this meta-analysis of hundreds Hb structures. See Materials and Methods (MM) for a description of the structural collection and a list of notations used in both articles. Readers may find a brief guide helpful from MM of the companion article that outlines the relationship between the diverse topics presented in these two articles.

## Results and Discussion

### Concentration of information

The ultimate goal of meta-analysis is a big picture of “the whole elephant” rather than biased perspectives of specific parts of the subject with limited scopes. Singular value decomposition (SVD; see Ref. [6] for a brief summary), a linear algebraic procedure, ranks the significance of information according to their consistency among multiple sources, thereby achieving a big picture via information concentration. SVD is widely applied in many disciplines of science for signal processing. It has been successfully adopted to analyze many related structures jointly [4], in particular, to process weak signal in crystallography [4,7,8]. Here I apply SVD analysis to a collection of static structures in PDB (MM; Figure S1) to extract structural dynamics. Only a small handful of *n* decomposed lower triangles of distance matrices (MM; Figure S2) are sufficient to reconstruct hundreds of *N* experimental structures using linear combination coefficient sets obtained from SVD. In other words, structural information is effectively concentrated with little loss of signal. Comparison with other methods of eigenanalysis is discussed in MM.

**Figure 2:**
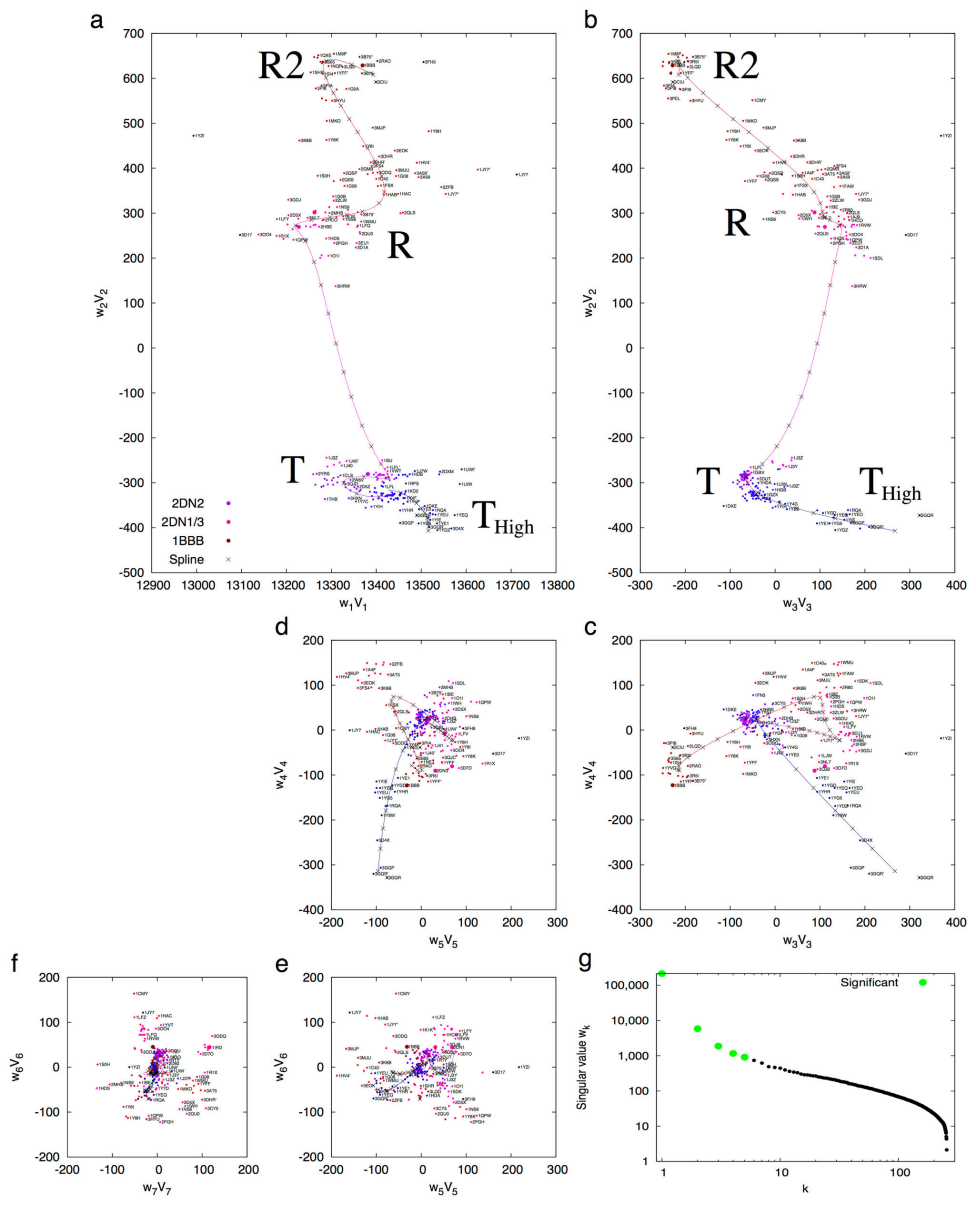
The first seven dimensions of Hb conformational space. The coefficient sets *w*_*k*_ ***V***_*k*_ with *k* = 1, …, 7 derived from SVD analysis of 280 tetrameric structures are plotted in **a** - **f**. Each pair of consecutive panels can be considered as two orthogonal planes by folding 90° along a line between them. The deoxy Hb 2DN2 is in blue. The oxy (2DN1) and carbonmonoxy (2DN3) forms of Hb are in red. The R2 state 1BBB is in dark red. Others are in continuous color transition from dark blue to blue to purple to red to dark red along the trajectory as a traveling salesman solution. PDB entries are labeled by small typeface whenever possible, and are only visible on a digital copy. The smooth trace through the multidimensional space is a spline fit to the traveling salesman solution that suggests a plausible reaction pathway T_High_-T-R-R2. Evenly spaced sampling points along the spline are marked by ×. The singular values *w*_*k*_ are plotted in **g** with the first five significant ones in green.

SVD analysis of distance matrices derived from 280 Hb tetramers results in five most significant singular values (Figure 2g). The first lower triangle U_1_ presents an averaged distance matrix similar to that displayed in Figure S2a. Figure S3 shows lower triangles U_2_ - U_5_ that are ranked second through fifth by SVD. The coefficient sets for linear combination *w*_*k*_ ***V***_*k*_ with *k* = 1, …, 7 are plotted in Figure 2a - f. It is clear that the tetrameric Hb structures cluster into several distinct groups. The group with tightest clustering consists of T state structures as represented by the deoxy wild type structure 2DN2 [9], suggesting more uniform T state structures than those in other states. The R state structures, as represented by the oxy (2DN1) and carbonmonoxy (2DN3) structures [9], exhibit less conformity in a group distinct from the T group and clustering much more loosely. Since T and R groups are separated along the second dimension *w*_2_V_2_, they mainly differ in composition of the second lower triangle U_2_ (Figure S3a), which bears good resemblance to the difference distance matrix between the oxy and deoxy structures (Figure S2b). The R2 structures [10], as represented by 1BBB, form a subgroup further away from T in the scatter plot of the first two dimensions (Figure 2a, b), although R2 is closer to T in other minor aspects [11] as suggested by the coefficients *k* = 3, 4, and 5. It has been shown that R2 could be a more representative state for ligated Hb in solution [12]. It is also noticeable that another subgroup stretching out from T consists of the structures in the high affinity T_High_ state (also known as B state [13]) that are often resulted from mutations at Trp37β [14]. Interestingly, several structures of the low affinity cat Hb [15] with intact Trp37β also belong to this group. While *k* increases to 6 and 7, all structures scatter in a fashion of normal distribution (Figure 2e, f), suggesting that these lower triangles U_6_ and U_7_ contain mostly to only noises. Elimination of the insignificant lower triangles removes inconsistent structural fluctuations in the structural collection, thus allows consistent functional motions to stand out. Figure 2 implies that each snapshot of Hb structure in PDB carries an order within a large collection, which is a source of dynamic information. The companion article takes advantages of this information implied by the order along a reaction trajectory to support the mechanism of Fe-Ni hybrid Hb and allosteric effectors [5].

### Reaction trajectory and the allosteric taboo gap

Many human Hb structures were interpreted as intermediates during the allosteric transition. However, subtle and inconsistent structural differences they present often raise doubts on the validity of the proposed mechanisms as Park et al. warned [9]. This meta-analysis approach aims at a more objective metrics to evaluate the progression of a protein structure along a reaction trajectory, and to identify a protein structure whether it is indeed an intermediate between two states, or whether it is along a reaction trajectory at all. Sorting of static Hb structures according to their coefficient sets in the conformational space of the first a few dimensions from SVD (MM) confirms that 1MKO indeed represents an intermediate structure between R and R2 states [16], however it clearly does not qualify as a unique state [9] (Figure 2a, b). This sorting also unambiguously identifies 1YZI as an outlying structure that does not agree with any other Hb tetramers (Figure S4). It is the most compact tetramer of all judging by the smallest value in the first dimension *w*_1_V_1_ (Figure 2a). 1YZI was previously assigned as a unique quaternary assembly R3, and hypothesized as a dead-end species [16]. However, the sole occurrence of such structure in the database leaves doubt on its functional relevance.

It is remarkable that the entire trajectory twists and turns several times along a spline curve (Figure 2), and is continuously sprinkled around with representative structures except a wide taboo gap between T and R states. This demonstrates that no Hb structure has ever been captured in middle of T-R transition despite decades of efforts with a variety of experimental strategies [17]. Numerous attempts to capture intermediate structures may have only influenced the structures towards the opposite states slightly. For example, 1IBE is a deoxy structure that would normally be in T state but it is trapped in R state [18]. 1GZX, on the other hand, is fully oxygenated but in T state instead of the usual oxy R state [19]. Both structures are clearly within the range of the “wrong” states in which they are trapped, judging by their locations in the reaction trajectory (Figure 2), although the presence and absence of ligand indeed cause some localized conformational changes towards where they should usually belong. By no means they represent the transition between T and R.

This global observation of allosteric taboo gap once again provides clear evidence for MWC allosteric theory that a Hb tetramer must be in either one of the two discrete states regardless of its status and extent of ligand binding [20]. Is the discreteness of states functionally required? If so, how does the structure achieve this discreteness? What structural basis has thus far strictly prevented any structure from being experimentally captured in middle of the T-R transition? It is conceivable that there must exist one or more meta-stable structures while the tetrameric structure traverses the wide allosteric taboo gap regardless how short-lived they might be [17]. It would be highly desirable to evaluate these hypothetical structures at atomic level.

### Back calculation of structures by distance geometry

SVD analysis on distance matrices in essence carries out structural comparisons at a large scale. Sorting of static structures according to their similarity as described in MM results in a trajectory in a multidimensional conformational space, along which the tetrameric Hb structure evolves from T_High_ to T to R to R2. A big picture of “the whole elephant” emerges as each experimental structure fits into it with respect to the rest of the PDB collection. This section presents a reverse process that constructs a 3-dimensional structure based on a linear combination coefficient set at any given location in the conformational space, which completes the analytical loop (Figure 1). Such back-calculated structures demonstrate atomic details of some hypothetical structures spanning the allosteric taboo gap that are otherwise not yet obtained by experimental approaches.

In the conformational space of the first a few significant dimensions, evenly spaced sampling points can be chosen along the smooth spline trajectory (Figure 2). A distance matrix is then recomposed from the first a few decomposed lower triangles ***U***_*k*_ (Figure S3) using the coefficient sets *w*_*k*_ ***V***_*k*_ corresponding to the coordinates of each sampling point. The resulting distance matrices evolve smoothly along the spline trajectory as shown in Movie S1.

Determination of a three-dimensional structure from a distance matrix is a previously solved problem known as molecular distance geometry, which is routinely used in nuclear magnetic resonance (NMR) spectroscopy (MM). Here I adopt the computational technique in NMR to solve “crystallographic” structures by a novel use of distance geometry. Each distance matrix in Movie S1 represents a hypothetical structure that is extracted from many experimentally observed structures. This numerical extraction of structural series in effect removes experimental fluctuations, thus warrants smooth motions, yet remains faithful to the experimental observations (Movies S2 and S3). Motions revealed by the back-calculated structural series are distinct from those derived from simple interpolation between two structures also known as molecular morphing [21]. Here the chief difference is the numerical removal of irregular structural fluctuations and other more advanced analyses in conformational space as discussed below. This technique provides a vital capability to identify the cause of functional motion as the experimental condition varies.

Ten back-calculated structures spanning the allosteric taboo gap (Figure 2) are distinct from any experimentally observed structures and provide structural insights into the molecular basis of the allosteric taboo gap. First, the well-known ratchet switches [22,23] at Cαi-FGβi (see MM for notation) requires either Thr38α or Thr41α in two consecutive turns of the 3/10 helix Cα to interact with FGβ at a proper position. In the back-calculated intermediate structures spanning the allosteric taboo gap by distance geometry, neither Thr38α nor Thr41α are positioned correctly to interact with FGβ.

Second, the β C-termini must have proper docking sites. In T state, the C-terminus of βi contacts Cαi, that is, two salt bridges of Perutz [22,24]. In R state, these β C-termini depart far from Cα and are now in contact with the β N-termini of their counterpart subunits. In R2, these β C-termini move even closer and eventually make contact with each other. In contrast, α C-termini largely hold a constant distance from each other. In the back-calculated structures spanning the allosteric taboo gap, the β C-termini are not properly docked but float in space amid the T-R transition (Figure S5).

Third, two pairs of H-bonds near the N-termini of Gs that involve two pairs of in-law subunits form and break alternately in T and R states. Two H-bonds Asp99βiO^*δ*^-Asn97αiN^*δ*^ form in T state. The equivalent residues in the in-law subunits form two other H-bonds Asp94αiO^*δ*^-Asn102βiN^*δ*^ in R state. Hb Titusville is a low affinity variant caused by Asp94αAsn mutation [25]. This mutant would replace the pair of strong H-bonds in R state with weaker H-bonds of either Asn94αiN^*δ*^-Asn102βiN^*δ*^ or even weaker Asn94αiN^*δ*^-Asn102βiO^*δ*^, which might explain that Hb Titusville favors low affinity T state. However, while the structure is crossing the allosteric taboo gap, none of the two pairs of H-bonds is intact.

Here the novel use of distance geometry presents a computational means to reveal plausible structures that may only transiently occur during allosteric transition. The more fundamental reason why these structures spanning the allosteric taboo gap have never been captured by crystallographic experiments is presented in the companion article [5], which is a direct outcome of the mechanism of quaternary transition crossing the wide taboo gap.

### Tertiary states

The tertiary two-state theory of Eaton et al. provides a major update to MWC, that is, oxygen binding affinity is dictated by the tertiary state of each subunit instead of the quaternary state of the tetrameric assembly [26,27]. Here I examine the tertiary states of more than 1,000 subunit structures available in PDB using meta-analysis. A relatively uniform tertiary t state for both α and β is clearly observed judging by tighter clustering of open circles in Figure S6. However, the tertiary r state consists of several sub-states, one of which for α is named r2, since they are only found in R2 quaternary state (Figure S6a, b). Another r sub-state of α is close to t, denoted r1. Only in the fourth dimension *w*_4_V_4_, r1 separates from t (Figure S6c). Similar sub-states of r also exist for β (Figure S6d, e, f). The main differences between t and r are localized at FGs and the C-termini of both subunits as seen from the second decomposed lower triangles (Figure S7a, d). These observations lead to the findings in the companion article that depicts the structural mechanism of intradimer cooperativity and quaternary rotation [5].

Nonuniform r is certainly insufficient to dispute the tertiary two-state theory, as R2 and T_High_ states do not invalidate MWC quaternary two-state theory. However, no subunit in t state has ever been found in R and R2 tetramers; nor r of any kind, including r1 and r2, ever exists in T tetramers as the linking gray lines demonstrate in Figure S6. In other words, t and r do not mix in a same tetramer as far as the current PDB presents (Figure S6). Furthermore, r2 does not coexist with other sub-states of r either. See discussion below on homotetrameric Hbs for potential exceptions. Again, MWC quaternary two-state theory prevails, and the rule of symmetry conservation is strictly obeyed.

All PDB entries analyzed here are static Hb structures, none of which features a mixture of t and r, because any such mixture in a tetramer can only exist transiently as predicted by the mechanism of the Hb machinery proposed in the companion article [5]. This remains true even for those Fe-Ni hybrid Hbs [28,29]. The tertiary two-state theory, however, was very successful in fitting time-resolved spectroscopic data [26,27]. The short-lived structural species that feature asymmetric mixtures of t and r, although crucial for understanding mechanism, do not accumulate to a significant concentration in blood nor in static crystals, since molecular events such as ligand binding, tertiary and quaternary transitions are not synchronized under physiological condition. Thus in a physiological time scale, the transient species are not required for satisfactory explanation of oxygen saturation data as MWC demonstrated [20]. Quaternary and tertiary two-state theories deviate only in the time scales of individual molecular events, when these events are synchronized by pulsed laser in time-resolved flash photolysis experiments [30,31]. Therefore, the tertiary two-state theory is in complete agreement with MWC, Pauling-KNF, the molecular code, and my analysis here and in the companion article.

**Figure 3:**
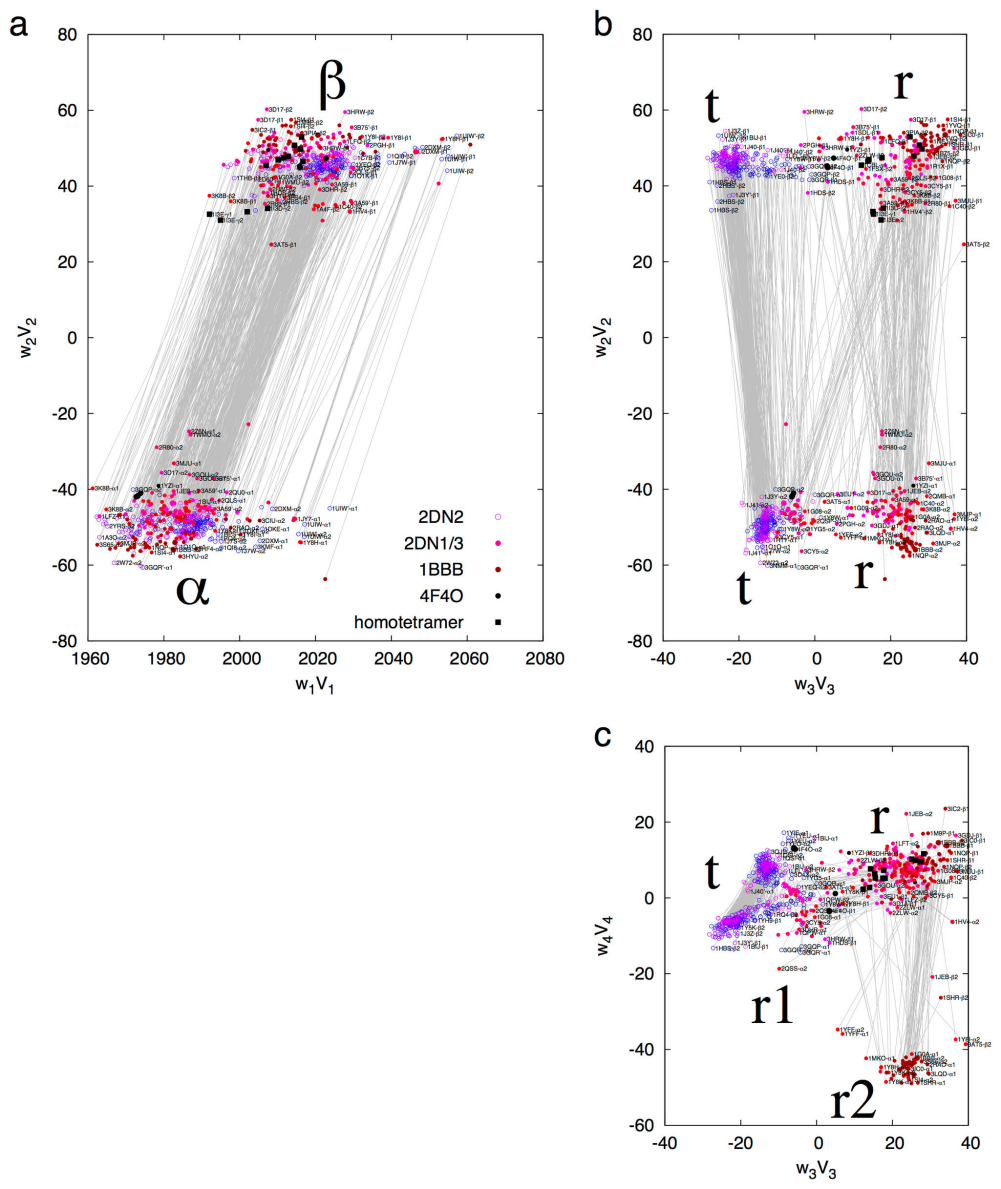
Combined conformational space of α and β. SVD is performed on more than 1,000 distance matrices of the equivalent residues of α and β. The 5-residue insertion in CDβ and 2-residue insertion in ABα are excluded in the calculation. The coefficient sets *w*_*k*_ *V*_*k*_ with *k* = 1, …, 4 are plotted in **a** - **c**. The continuous color scheme is the same as in Figure 2. In addition, all subunits on T side of the allosteric taboo gap are represented by open circles, and those on R side are in solid dots. Four subunits from the structure of free dimers (4F4O) are in black dots. Those β and γ subunits from the abnormal homotetrameric Hbs are in black squares. Two subunits from a same dimer are linked by a gray line. PDB entries are labeled by small typeface whenever possible, and are only visible on a digital copy. The decomposed lower triangles are in Figure S8.

SVD analysis is jointly applied to the equivalent sections of α and β, in which several small insertions in both chains are excluded. Therefore, their conformational spaces separately presented in Figure S6 are combined in Figure 3. The second dimension presents the major difference between two chains (Figure 3a, b). The third dimension shows that both subunits share common difference between their two tertiary states (Figure 3b). The gray lines in Figure 3 that connect α and β in a same dimer do not cross over between t and r states. Some apparent crossovers in Figure 3b only reach the r1 state that is close to t of α as better observed in Figure 3c. This observation of symmetry in tertiary states is interpreted in the companion article as any change in one subunit is reciprocated by the symmetrical change in the partner subunit. This clearly shows the effectiveness of the intradimer mechanical coupling via the lever system of helices at the dimeric core [5].

SVD analyses of two globin chains jointly and separately also mimic what might be the outcome when other structural collections from different organisms are analyzed by SVD. This example shows that SVD is capable of isolating major structural difference caused by sequence variation.

**Figure 4:**
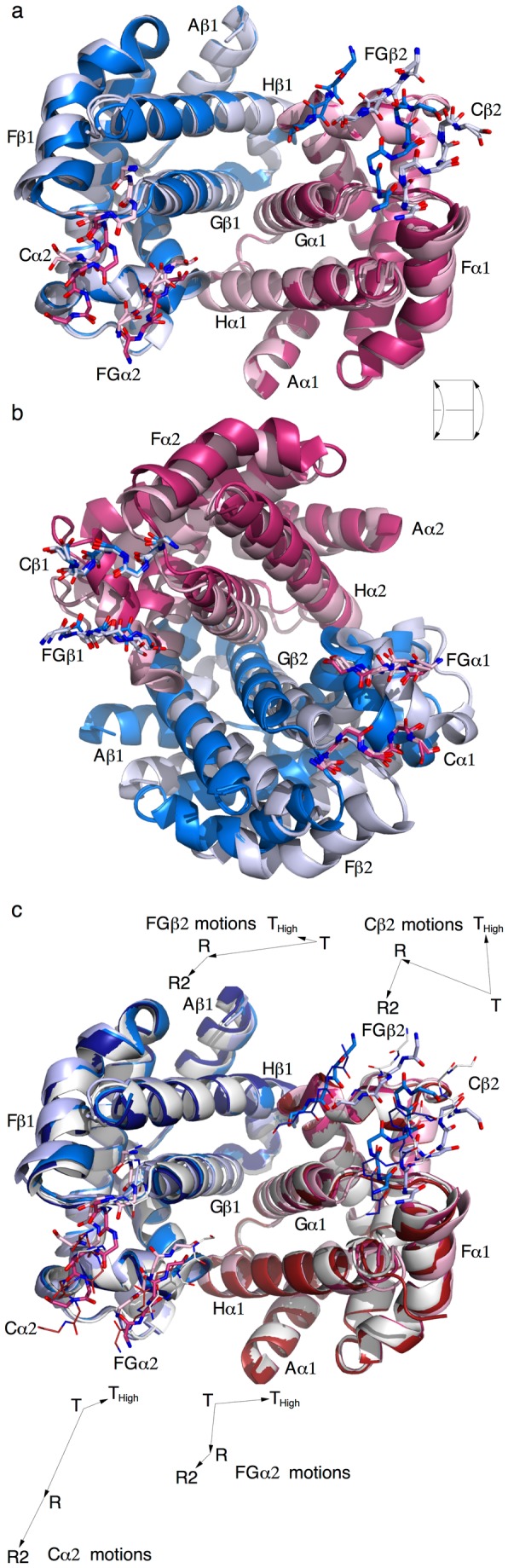
Back calculated structures by distance geometry. α and β are in warm and cool colors, respectively. Quaternary T state is in light pink and light blue. R is in median red and median blue. R2 is in dark red and dark blue. T_High_ is in white. A ribbon model represents one dimer in background. The opposite dimer is then in stick model in foreground. T and R are in thick stick models. T_High_ and R2 are in thin stick models. All structures are aligned by least-squares fitting of the invariant framework of α1β1 identified by the rmsd matrix (Figure S11a). **a**. Five structures are superimposed: Ttttt or 01, Trttt or 11, Trrtt or 21, Rrrrt or 31, and Rrrrr or 41. α1β1 is in background and α2β2 is in foreground. Movie S4 shows these structures in motion. **b**. Same as **a** except that α2β2 is in background and α1β1 is in foreground. The relation of viewpoints is indicated by a flip of the page. Movie S5 shows these structures in motion. **c**. Same view as in **a**. Four consensus structures are superimposed: T_High_, T, R, and R2. Some motions of α2β2 are also indicated by the inserted vector diagrams. The lengths of vectors are twice as long as the observed motions.

Transient structural species of human Hb that would demonstrate a working cooperative machinery are yet to be captured by time-resolved crystallography. However, we have recently reported a structure of an invertebrate dimeric Hb in an asymmetric state that occurs 100 ps after photodissociation of a CO ligand. This transient structure is shown responsible for motion transmission between two subunits [4] as a crucial intermediate that the sequential model of Pauling-KNF requires [32]. The reaction trajectory of human Hb derived here from static structures (Figure 2) does not take into account of the transient species of asymmetric states, and thus is still an oversimplification at the crossing of the allosteric taboo gap (Figures 2 and S5). A more realistic trajectory with twists and turns in this crossing section is expected according to the microstates and tertiary two-state theories. To further explore the atomic details during tertiary and quaternary transitions, distance geometry is again used to model the transient species 11 or Trttt, 21 or Trrtt, and 31 or Rrrrt (Figure 4a, b). For example, the distance matrix of Trttt is a composite of those of Ttttt and Rrrrr. The only triangular portion of α1 intra-subunit distances of Ttttt is substituted with the same triangle in Rrrrr. In such composite matrices, intra-subunit triangles are determined by the tertiary states while inter-subunit rectangles are determined by the quaternary states; and potential geometric conflicts are resolved by distance geometry. The five structures Ttttt or 01, Trttt or 11, Trrtt or 21, Rrrrt or 31, and Rrrrr or 41 displayed in Figure 4a, b, Movies S4 and S5 show that upon the first ligand binding, the pinch motion of two FGs starts to bring them closer towards each other, which subsequently drives the quaternary rotation, and fully develops after the quaternary rotation completes [5]. This molecular modeling method based on distance geometry applied to composite distance matrices further extends the capability of meta-analysis (Figure 1) to determination of hypothesized structures via hybridization of experimental structures.

### Helix identification matrix and helical transformation

**Figure 5:**
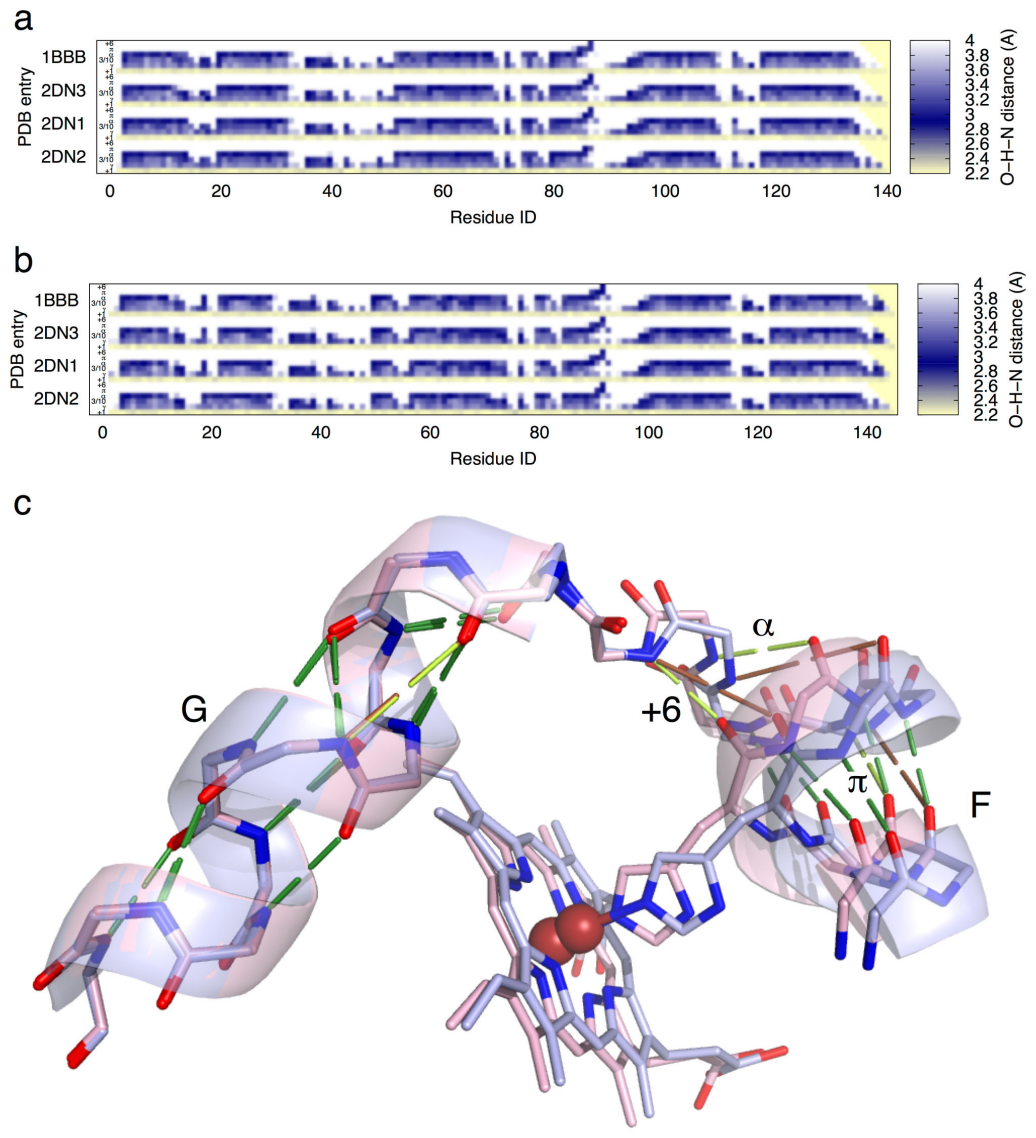
Main chain H-bonds. Upon ligand binding, the main chain around heme anchor gains a total of four H-bonds. However, these additions do not occur simultaneously, and they take place differently in α and β. a. HIM of α. b. HIM of β. An HIM is a specialized distance matrix that contains only helix-forming H-bond lengths in a main chain, and has only several rows as labeled +1, γ, 3/10, α, π, and +6. The *i*-th column contains distances from a main chain carbonyl O of residue *i* to the amide Ns of residues *i* + 1 to *i* + 6. A distance value in a row within the proper range for H-bonding is colored in dark blue to show strong H-bond and to indicate a specific type of helix or turn. Large distances fade to white and short distances turns into yellow. PDB entries are labeled on the left. c. Main chain consensus around heme anchor in all states and subunits. T state structure is in light blue. A ligated state in R or R2 is in pink. The C-terminus of F that anchors the heme group is shown on the right. All side chains except the proximal His are removed. The constant H-bonds in all states are presented by dark green rods. The newly formed H-bonds in ligated states are in light green. These H-bonds not yet formed in T state are in brown. The strong H-bond gained at proximal His87αO-Val93αN is marked +6. The equivalent H-bond in β proximal His92βO-Val98βN already exists in T state. A weaker H-bond Ala88αO-Arg92αN continues the α conformation as labeled α. In R2 state, an additional strong H-bond Asp85αO-Lys90αN extends the π helix further upstream. In β, the equivalent new H-bond Glu90βO-Lys95βN is the only addition that extends the π helix upstream in both R and R2 states as labeled π. A weaker addition of H-bond in G on the left is Pro95αO-Lys99αN, which puts the beginning of Gα in a transition between 3/10 and α conformation. This transitional conformation is constant in β.

**Figure 6:**
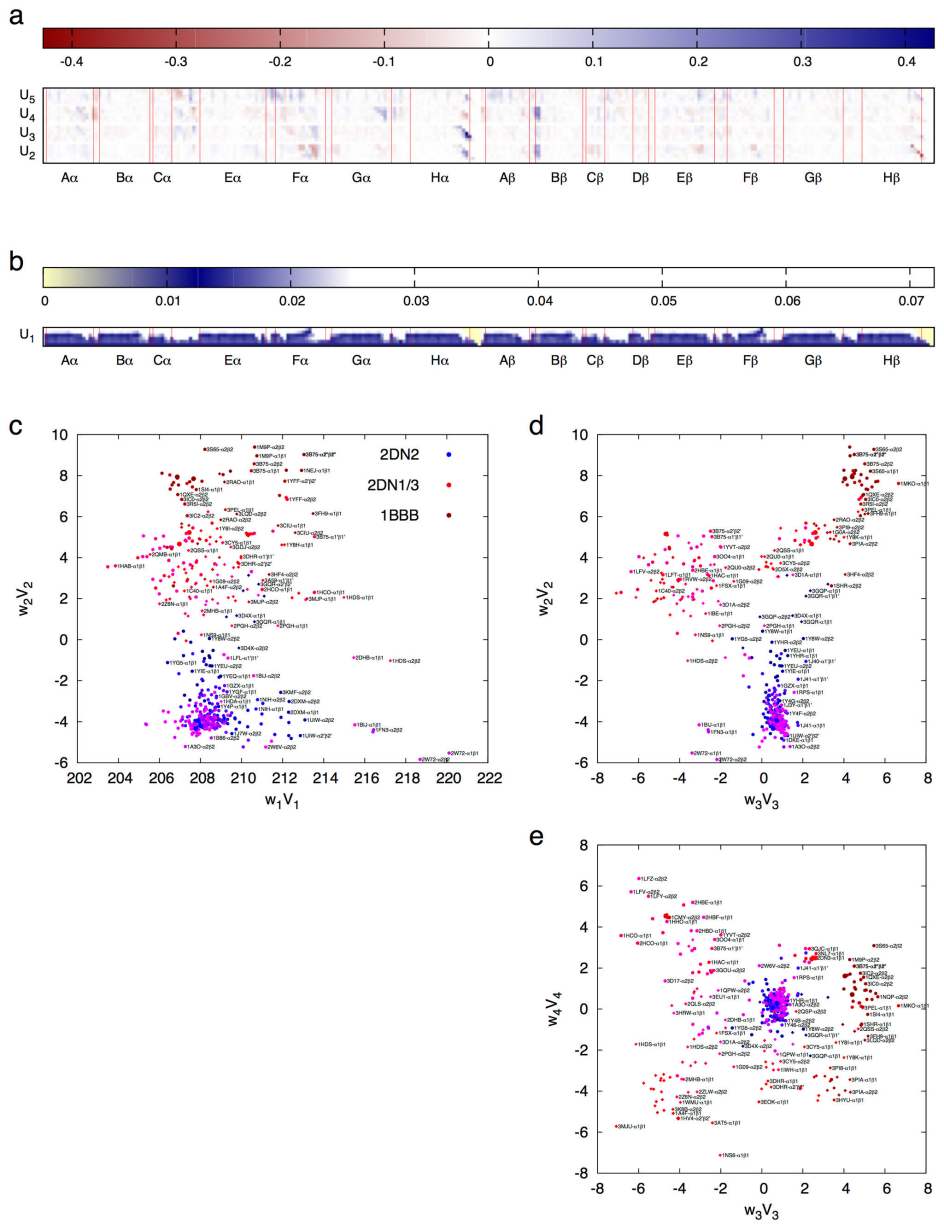
SVD analysis of HIMs. SVD analysis is applied to HIMs of 560 αβ dimers in the structural collection. **a**. Left singular vectors U_2_ - U_5_. **b**. Left singular vector U_1_. This can be considered as the average of all 560 HIMs. **c** - **e**. Scatter plots of the first four dimensions of SVD. For example, each value of *w*_2_V_2_ indicates how much composition of U_2_ is required by an HIM. Dimers are labeled by small typeface as much as possible if the labeling is not interfering with other graphing, and are only visible on a digital copy. The continuous color scheme is identical to that in Figure 2. Structures of human and non-human Hb are indicated by solid dots and diamonds, respectively.

The analytical strategy presented above that constructs an overall conformational space (Figure 1) can be further extended to partial distance matrices to address specific issues of structural variation. Here I design a specialized distance matrix for identification of several types helices in a protein structure. Joint analysis of helix identification matrices (HIM; Figures 5 and S9) using SVD not only identifies various helical formations such as 3/10, α, and π helices, but also reveals unusual features as a helix unwinds or undergoes transitions between different types as a reaction progresses. Four most significant changes in main chain H-bonds of human Hb are revealed at both C-termini of α and β, around F and FG in α, and at the beginning of Bβ, where large positive and negative values are observed in the right singular vectors U_2_ - U_4_ (Figure 6).

The C-termini of both α and β are essential for proper transmission of motions originated in the allosteric cores to the distal blocks of the partner subunits (Figure S10) [5], which is reflected by the changes of the main chain H-bonds in these C-termini. SVD analysis of HIM identifies that during T-R transition, the C-terminus of α becomes extended, and even more so in R2. As a result, a 3/10 H-bond Thr137αO-Tyr140αN is lost (Figures 5a and 6a). In contrast, two H-bonds Ala142βO- and His143βO-Tyr145βN in the C-terminus of β newly forms in R and R2 states (Figures 5b and 6a), which swings two C-termini of the counterpart βs towards each other and eventually makes contact between them in R2 (Figure S5 and Movie S2).

The concerted motions of F and G upon ligand binding in the crowded distal environment result in a large pinch motion of two FGs towards each other [5]. Significant changes in main chain H-bonds at the C-terminus of Fα to the N-terminus of Gα accompany this largest motion observed within a dimer (Figure S11a; see also Movie S1 of the companion article [5]). However, the equivalent changes of H-bonds occur to a lesser extent in β [14] (Figure 6a). Both heme anchor sites are in π helices as noted previously [14,23], which is confirmed by strong H-bonds in the π row of HIM (Figure 5a, b). Between proximal His87αO and Val93αN, a strong +6 H-bond is gained upon ligand binding (Figure 5c). Overall, β carries more main chain H-bonds around the heme anchor site in T state and gains one more upon ligand binding, thus β represents a more advanced conformation along the reaction trajectory towards R and R2 states. On the other hand in α, four additional H-bonds are gained in the equivalent region as the reaction progresses towards the final R2 state, but these new H-bonds form in two separate steps (Figure 5c). A mechanical model of cooperative oxygen binding presented in the companion article illustrates that these additional H-bonds are directly responsible for preventing motion backlash and transmitting ligand induced motions to generate the large pinch of FGs, which subsequently drives both intradimer cooperativity and quaternary rotation [5].

The third remarkable change revealed by the analysis of HIMs occurs at the first turn of Bβ, which is in intact α helix conformation in T state but pried open in R and R2 states as the main chain carbonyl groups of Val20β and Asp21β flip outward (Figure S12b, c). These two residues are located one turn ahead of the crossing point of two helices Bβ and Eβ (Figure S13b), a key structural feature that conveys rigidity of the dimeric structural core to E on the molecular surface [5]. In contrast, the first turn of Bα remains in constant α conformation in all states (Figure S12a). Flexibility in the first turn of Bβ of human Hb may be necessary to sustain the dovetailed helix crossing next to this turn, while α has a two-residue insertion of Gly16α and Ala17α in ABα (Figure S12a) that would play a similar role, thus such change of H-bonds is absent in α. It is also noticeable that only human Hb features such change of Bβ (Figure S12d). The other tetrameric Hbs tend to have negative coefficient *w*_4_V_4_ while this coefficient is mostly positive for human Hb (Figure 6e), that is, U_4_ cancels out U_2_ at the beginning of Bβ (Figure 6a) in non-human Hbs. Apparently, the first turn of Bβ can be interpreted as a highly refined mechanical cushion that maintains the integrity of the critical Bβ-Eβ crossing (Figure S13b) as human Hb undergoes large conformational changes from state to state [5]. Alternatively, the integrity of Bα-Eα crossing (Figure S13a) is protected by a two-residue insertion in ABα (Figure S12a).

### R2, T_High_ states, free dimer, and homotetramers

Relatively late discoveries of R2 [10] and T_High_ [14] quaternary states are considerably more demanding for allosteric theories to formulate unified explanations. Structural mechanisms that interpret these additional quaternary states face even greater challenge. Figure 4c shows a superposition of the consensus conformations in the four quaternary states. The only significant motion within a dimer is the pinch motion of two FGs. T_High_ exhibits the largest amplitude of pinch, which is consistent with the averaged distances between FGs directly calculated from the Cartesian coordinates [5]. SVD analysis of dimer structures also reveals that dimers in T_High_ state, especially those from cat Hb (3D4X, 3GQP, 3GQR, and 3GYS), bear close resemblance to those in R state in the conformational space of dimer (Figure S14a, b). Therefore, all evidences presented here and in the companion article lead to a same conclusion, that is, the lever system of Hb dimer is built to accomplish a large pinch motion [5], and this motion is largely identical in R, R2, and T_High_ relative to T except slightly different extensiveness. How a dimer would respond to the pinch motion of the opposite dimer differentiates the quaternary states. T-T_High_ quaternary transition is often found in mutants near the flexible hinges, most noticeably Trp37β in Cβ and Tyr140α [14]. These mutations weaken the hinges and cause the separation of the flexible hinges Cβi-FGαi in T_High_ state. The motions of separation in T-T_High_ transition are almost perpendicular to the motions in T-R transition (inserts of Figure 4c). In the mean time, the normal ratchet switches Cαi-FGβi become relatively stronger than the weakened hinges and turn into hinges so that the ratchet motions in T-R transition are absent in T-T_High_ transition (Figure 4c). Therefore, T_High_ state is resulted from a reversal of the roles that the flexible hinges and ratchet switches would normally play. The high affinity in T_High_ state is again caused by the large pinch of FGs just as such motion results in high affinity in R state [5]. It would be highly intriguing but is presently unclear whether cat Hb would use T-T_High_ branch of the reaction trajectory to achieve ligated state instead of the usual R state as some preliminary results indicate that the ligated cat Hb is located at one end of the reaction trajectory in T_High_ state (3D4X, 3GQP, 3GQR, and 3GYS in Figure 2).

In the recent crystal structure of a haptoglobin-Hb complex (4F4O), two Hb dimers are separated from each other by a haptoglobin dimer [33]. These free Hb dimers assume a typical conformation of R state (Figure S14), in which β is in a typical r state (Figure S6d, e, f) and α is closely similar to r1 (Figures 3 and S6c). This is not surprising since the dimers are in the oxy form. I wish to predict that a deoxy form of the free dimer would not move into T state, since T state is only achievable by bending two Gs outward thus lengthening an extra 3 Å in the distance between two FGs and clamping the jaws onto two fixed Cs of another dimer. When two dimers clamp onto each other, the oxygen binding affinity in all four subunits drop significantly [5]. Thus a free Hb dimer would not reach low affinity T state regardless of its ligand binding status. A constantly high affinity free dimer is non-cooperative [34], since ligand dissociation would not trigger changes in the lever system as Gs would not be bent without Cs from an opposite dimer to cause the motion.

Finally, understanding the molecular mechanism of tetrameric human Hb may have far-reaching medical implications. The new findings presented in this and the companion articles directly shed light on the abnormal homotetrameric Hbs. A long-standing question is why α and β chains differentiate in tetrameric Hbs. This is apparently required by the mechanism of T-R quaternary transition. The residues in Cβ equivalent to Thr38α and Thr41α in Cα are much larger Trp37β and Arg40β. These large side chains prevent FGαi from sliding on Cβi unlike the smaller Thr side chains in Cα that function as notches. If Hb were assembled from four chemically identical chains, the functions of two hinges and two ratchets at four interdimer couplings would not differentiate. Such homotetramer occurs in α-thalassemia, a frequent genetic trait in some human populations that may alleviate the impact of malaria [35]. As some or all α globin genes are missing, insufficient α chains lead to excess β chains to form β_4_ HbH. In most severe case during fetal development, γ chains that are equivalent to adult β chains assemble into γ_4_ Hb Bart’s that causes stillbirth or newborn death [35]. Due to non-cooperative high affinity, HbH and Hb Bart’s have diminished ability to transport oxygen. Without Thr residues in Cα, the normal ratchet mechanism is lost, and all four interdimer couplings are constantly locked by the large side chains in Cβ. Therefore no quaternary rotation can occur, nor can cooperative binding. Any ligand-induced change in the interdimer couplings would have equal chance at each of the four sites, and a likely change would be similar to the separation of hinge found in T_High_ state [14]. Joint SVD analysis with the β or γ subunits in homotetramers shows that the tertiary structures of both deoxy (1CBL) and ligated β (1CBM) or γ (1I3D and 1I3E) subunits are in typical r state (Figure 3), which illustrates the reason for high affinity with or without ligand and validates the tertiary two-state theory. However, all dimers in homotetramers are located between T and R states but closer to T state in the conformational space of dimer (Figure S16). Ligand binding moves their conformation only slightly towards R state. The homotetrameric assembly was described as R-like [36–38]. The typical tertiary r state, the nearly dimeric T state, and the R-like quaternary state seem contradictory to one another. This indicates that the jaw of two FGs in a homotetramer is open wide enough (dimeric T) to reach Cs even when ligands are bound and Gs are straight (tertiary r), which explains the unstable HbH and Hb Bart’s. Ligand binding causes no pinch of FGs, no quaternary rotation [37], thus no cooperativity.

## Materials and Methods

The methodological advance in this work is an effective implementation of a large-scale structural comparison in a conformational space with a manageable dimensionality. This approach circumvents structural alignment and returns back to Cartesian real space via a novel use of molecular distance geometry, the chief algorithm in NMR. In contrast to pairwise structural comparison that is more susceptible to random structural variation, this conformational space achieved by SVD analysis of distance matrices unifies a diverse structural collection. Inconsistent structural fluctuations due to source of organisms, mutants, crystal forms, data quality, and many experimental details is identified and arranged in higher dimensions of the conformational space. Isolating the common trend of structural motions from these fluctuations clarifies the functional dynamics of Hb among profuse structural variations less relevant to the function.

Protein conformational space is generally considered as an immense space with high dimensionality [39,40]. If a sufficiently large collection of related structures is available, the accessible conformational space has been experimentally mapped out to a certain extent. It has been previously shown that the dimensionality of this space needs not to be large, if each dimension represents a synthetic variable or collective coordinate [39]. Here the decomposed lower triangles (Figure S3) provide an alternative implementation of the common principle in eigenanalyses such as principal component analysis (PCA). This implementation of simultaneous comparison of a large structural collection first takes advantage of the drastic reduction in dimensionality given experimental structures in PDB. Secondly, the findings from this simultaneous comparison conversely explain why the dimensionality could be reduced to a small number. For example, an αβ dimer can only perform one motion despite of four different quaternary states – the intrinsic pinch of two FGs that is coupled by the lever system at the interface between subunits. Therefore, one dimension is sufficient to describe this motion, that is, *k* = 2 if α and β are considered separately (Figure S7a, d), *k* = 3 for a consensus of both chains (Figure S8b), or *k* = 2 in the conformational space of the dimer (Figure S15a). Similarly, the tetrameric assembly is capable of one motion, the quaternary rotation. Because of the change of rotation axis in three laps of the quaternary transition, two or three dimensions (*k* = 2, 3, and partly 4 in Figures 2b, c and S3a - c) are sufficient to quantitatively describe these rotations. Here, an important implication on protein structure-function relationship is that each protein structure is designed or evolved to accomplish one specific function. Functional specificity of protein greatly restricts its accessible conformational space to a very confined dimensionality. Thus this meta-analysis technique presents an example of post structural genomics approaches to study protein dynamics using the cumulative experimental data that become increasingly abundant in PDB.

### Notations

The following notations are consistently and extensively used throughout this and the companion articles [5]. Whenever possible, “helix E” or “matrix **A**” are referred as single letters “E” or “**A**”.

α, β, γ   Globin chain types.

α_2_β_2_   The heterotetrameric assembly of Hb. Subscript integers are used as those in a chemical formula.

αβ   The heterodimer.

α1, α2   Subunits. Integers in normal typesetting are used to distinguish two chemically identical chains.

α1′, β2″   Subunits. An asymmetric unit of some crystal structures contains more than one tetramer. Prime (′) and double prime (″) are used to mark subunits from the second and the third tetramers in some crystal lattices.

2DN1   A four-character alphanumerical string is a PDB entry. It may also represent the tetramer from that entry. Prime (′) and double prime (″) appended to an entry indicate the second and the third tetramers in some crystal lattices. Dot (.) appended to an entry indicates a tetramer with α1β1 and α2β2 swapped.

E, F   Helices. Helices A through H are denoted by single letters.

Eα, Fβ2   Helices in specific subunits.

FG, CEα1   Corner or loop between two helices.

E7, DE2   Residue positions in helix or loop.

His92β   A residue in a chain.

C^*α*^,S^*γ*^   Atom type and position, to distinguish from Cα, helix C in subunit α.

*n, K*   Variables in italic.

*V*   Vector in italic bold.

**A**   Matrix in upright bold.

“Partner” subunits refer to α and β in a same dimer. “Counterpart” subunits refer to two α subunits or two β subunits in a tetramer. “In-law” subunits refer to two subunits of different chains from different dimers, that is, α1 and β2 or α2 and β1, which are denoted as αi and βi for conciseness.

### Structural collection of tetrameric Hbs

Meta-analysis is intended to evaluate large structural collections that differ from static ensembles of protein structures. For example, a bundle of structures resulting from an NMR experiment represents a typical static ensemble. A static structural ensemble does not include a variable condition that is responsible for the structural variation within the ensemble. The structural variation exhibited by a static ensemble may be stretched to infer functional dynamics. A structural collection subjected to the meta-analysis presented here is assembled from hundreds of Hb structures in PDB, each of which is associated with a detailed experimental condition. One of the chief purposes of meta-analysis is to identify the structural cause of a functional motion. Thus the ability to track the experimental condition of each structure in the collection is indispensible.

Human Hb [41] and other mammalian, avian, and reptile Hbs are included in the structural collection. Some residue IDs are changed to follow the standard of human Hb. Wild types and all available mutants are included. The collection contains 280 tetramers from 264 PDB entries. Their crystallographic resolutions range from 1.07 to 4.5 Å. Figure S1 shows the history of PDB entries for tetrameric Hb. An acceleration of deposition occurred since 1992 until 2005. Currently, Hb entries are still increasing at a rate of 15/year judging by the records in the recent years.

The global analytical strategy developed here is designed to isolate the consistent structural motions from structural fluctuations caused by all possible reasons, for example, source such as sequence variations and mutants, experimental conditions such as pH and temperature, and data quality such as resolution limit and refinement quality. Low resolution structures carry more signal than noise into the global analysis. The noise that is inconsistent with the structural collection will be isolated by the analytical method.

### Distance matrix, difference matrix, and rmsd matrix

Distance matrix contains pairwise distances from all atoms, main chain atoms, or only C^*α*^ atoms of a structure. All items on the major diagonal of the matrix are zero. Since the upper and lower triangles are symmetric, only the lower triangle is presented. An everyday example of distance matrix is an intercity mileage chart. A lower triangle calculated from *K* atoms contains *M*=*K*(*K*−1)/2 items. A distance matrix faithfully represents an atomic structure without a coordinate system, thus the structure is extracted from its crystal lattice without information loss other than its position and orientation in the lattice. A distance matrix is in fact a redundant representation of a Cartesian coordinate set. It can be shown that the redundancy is (*K*−1)/6 , thus analyses based on distance matrix could be quite expensive in computational time and storage for large structures – a major disadvantage of distance matrix. The average value of each column (or row) indicates the average distance of an atom to all other atoms, which provides a measure of interiority of an atom. The distance matrix of tetrameric Hb is shown in Figure S2a as an example.

A difference matrix contains the difference of the two corresponding values from two distance matrices of the same size [11,18]. The column (or row) average of a difference matrix measures the amplitude of structural difference at a specific atom. Figure S2b shows an example of difference matrix between carbonmonoxy (2DN3) and deoxy (2DN2) forms of Hb. If the difference is expressed in percentage, the relative difference matrix is also informative (Figure S2c).

Figure S2d shows an example of rmsd matrix calculated from 280 tetrameric Hb structures. Rmsd matrix can be used to identify mobile sections of the structures and relatively invariant structural frameworks (see MM of the companion article [5]).

### Singular value decomposition

The previous applications of SVD in crystallography were applied to (difference) electron density maps under the assumption that structural changes are localized in real space and noise are randomly distributed [4,7,8]. Here I further develop SVD application to jointly analyze a large number of related structures that have been previously refined and serve as the best available atomic models of their electron density maps, such as those archived in PDB. However, neither a PDB entry expressed in form of Cartesian coordinates nor its electron density map would be ready for SVD procedure without structure alignment, since they are only valid with respect to a specific coordinate system related to the original crystal lattice [3,42]. Choice of a suitable alignment protocol is often tricky [43] and all protocols may introduce artifact to the result of SVD. Here I choose to use pairwise inter-atomic distance matrix [44], and in some cases squared distance matrix [45], to represent a protein structure instead of electron densities or Cartesian coordinates of atoms. Although a standalone distance matrix does not seem informative to the eyes (Figure S2a), identification of structural change by the difference of two distance matrices (Figure S2b) does not rely on structural alignment. This major advantage of distance matrix over electron densities and Cartesian coordinates makes it directly suitable for SVD analysis, because it is global, lossless, coordinate-free, and independent of crystal lattice. Nevertheless, it is worth to point out that distance matrices of structures with sequence deletions and insertions cannot be compared directly. Such comparison would involve sequence alignment and exclusion of insertions. See Figure 3 for an example.

Each distance matrix (only its lower triangle is sufficient) calculated from a set of atomic coordinates is linearized and assembled into a column of length *M* in a data matrix **A**. *N* related structures make up the rectangle *M* × *N* data matrix, where *M* >> *N*. All *N* distance matrices are of an identical size. SVD factorizes matrix **A** so that

$$A=UWV^{T}$$ (1)

Matrix **U** has the same shape as **A**, and contains *N* orthonormal left singular vectors ***U***_*k*_ of length *M* with *k* = 1, …, *N*, which can be restructured according to the inverse protocol of linearization to form decomposed lower triangles. Since each ***U***_*k*_ has a unit length, the average value of the elements in ***U***_*k*_ is

$$\langle u\rangle =1/\sqrt{M}$$ (2)

The *n* significant lower triangles identified by the greatest singular values *w*_1_ through *w*_*n*_ on the major diagonal of matrix **W** can be used in a linear combination of ***U***_*k*_ with *k* = 1, …, *n* to reproduce a distance matrix that closely resembles the original one calculated from a structure and stored in a column of **A**, where *n* < *N*. That is to say, structural information distributed over all *N* distance matrices in matrix **A** is now concentrated in the top *n* decomposed lower triangles.

The coefficient set of the linear combination is *w*_*k*_ ***V***_*k*_ with *k* = 1, …, *n*, where each ***V***_*k*_ is a right singular vector in a column of matrix **V** or a row of the transposed matrix V^T^, and contains the relative compositions of the decomposed lower triangles ***U***_*k*_. Each coefficient set *w*_*k*_ ***V***_*k*_ for reproducing a distance matrix can be presented by a dot in an *n*-dimensional space. A graphical rendering of this multidimensional space on paper takes a form of multiple projections onto several two-dimensional subspaces (Figures 1 and 2). Therefore the coordinates of the dot indicate the compositions of the significant lower triangles ***U***_*k*_, also known as synthetic variables or collective coordinates. If two data points are close to each other in this *n*-dimensional space, they represent similar compositions, thus these two distance matrices are alike, so are these two structures. If two data points are far apart, these two distance matrices require very different compositions of ***U***_*k*_, thus these two structures are distinct. As a conclusion, SVD analysis of distance matrices provides an effective means to cluster related structures according to their similarity. More importantly, when structures are dissimilar, they may differ in various aspects. SVD analysis of distance matrices quantitatively describes these aspects in orthogonal dimensions.

### Conformational space

The role of the scatter plots displaying coefficient sets (Figure 2) can be related to Ramachandran plot [46], a distribution of main chain dihedral angles ψ and *ϕ* in the conformational space of amino acids. The conformational space of an entire main chain could be defined by all of its dihedral angles along the backbone. But this definition is not practical because of its large dimensionality. The SVD analysis on distance matrices presented here effectively reduces the dimensionality [39] to a small, manageable number (*n* = 5 in case of tetrameric Hb). Here I define this *n*-dimensional space as the conformational space of the protein structure under investigation. Since the dimensionality is so much reduced, each dimension is no longer a variable as simple as a dihedral angle. Instead, a large amount of structural information constitutes each of the *n* dimensions as presented by the significant lower triangles (Figure S3), that is, a synthetic variable or collective coordinate [39]. Substantial concentration of structural information into a small number of significant lower triangles by SVD drastically reduces the dimensionality of a conformational space. Just like a Ramachandran plot that shows the conformational distribution of all residues in a polypeptide chain, the scatter plots of SVD coefficient sets display the conformational distribution of all structures in the collection.

The scattered dots in the conformational space representing the coefficient sets can be sorted into a sequence. This sorting is a traveling salesman problem, a benchmark of combinatorial optimization, that is, to find a shortest route passing each data point once and once only [47]. This is to imply that the structural transition along a plausible reaction trajectory shall be as smooth as possible. A slight addition to the problem is to identify a home data point as the start of the route so that a one-way solution has the shortest path length. That is to say, the two end states shall be as distinct as possible. This computational problem can be solved by the simulated annealing algorithm [48]. Although the solution is not guaranteed to be the global minimum, it is likely to be one of the best solutions. A second addition to the traveling salesman problem is that a solution of reaction trajectory may bypass some structures that do not necessarily lie on the trajectory. Identifying outlying structures is also informative. An easy identification is to evaluate the saving in the length of the route gained upon bypassing a certain data point (Figure S4).

An even better representation of the reaction trajectory is likely to be a multi-dimensional spline fit to the traveling salesman route. The spline curve does not have to pass any data point but represents the consensus of all structures (Figure 2).

### Molecular distance geometry

Any given point on the spline curve represents a complete distance matrix that might differ from all experimental distance matrices. Structure determination from a distance matrix is a solved numerical problem. Nuclear magnetic resonance (NMR) spectroscopy routinely uses interproton distances accurately measured from nuclear Overhauser enhancement (NOE) effect [49] to determine protein structures. A number of mature software tools are available to perform distance geometry effectively, for example, CNS [50]. However, NOE effect is distance dependent and only close distances feature NOE. Here a full distance matrix imposes more numerous and more definitive restraints on a structural solution.

Since the underlying algorithm of the software that performs distance geometry has been well developed to incorporate energy minimization and stereochemical restraints [50], a back-calculated structure using such software does not only satisfy a distance matrix, but is also energetically and stereochemically plausible. Such back-calculated structures should be considered as hybrids of experimentally observed structures and theoretically modeled ones. In other words, they are molecular models highly restrained by experimental observations.

### Comparison with principal component analysis

In summary, the underlying principle of the reduction of dimensionality here is identical to that of PCA [3,39,51,52]. However, SVD applied directly to distance matrices retains key information in the conformational space, while circumventing structural alignment required by calculation of covariance matrix in PCA. Therefore, SVD is a far more versatile procedure than PCA. This is also exemplified in the application of SVD to HIMs. Distance geometry applied to recomposed distance matrices completes the analytical loop (Figure 1), so that the results of simultaneous comparison among a large structural collection by traveling salesman sorting in the conformational space can be evaluated in real space with atomic details. These new analytical capabilities have far exceeded that of PCA.

## Supporting Information

Figure S1 **History of Hb entries.**
(TIFF)

Figure S2 **Distance matrix (**a**), difference matrix (**b**), relative difference matrix (**c**), and rmsd matrix (**d**).** **a**. Distance matrix of the tetrameric deoxy Hb (2DN2). Four triangular portions of the matrix on the major diagonal contain intra-subunit distances. Each (nearly) square portion of the matrix contains inter-subunit distances. Column mean is plotted on top. **b**. Difference distance matrix. Difference is calculated between distance matrices of carbonmonoxy Hb (2DN3) and that in **a**. Positive and negative values are indicated by blue and red colors as shown in the color bar on the right. Small difference values are shown in pale green. The darkness of colors indicates that intra-dimer distance changes are greater than intra-subunit changes, but smaller than inter-dimer changes. All inter-subunit squares for partner and counterpart subunits are quite symmetrical about their major diagonals, such as α1-β1, α2-β2, α1-α2, and β1-β2. However, two inter-subunit squares for in-law subunits αi-βi are completely asymmetric. The symmetry of an inter-subunit square reflects the symmetry of the relative motion between these subunits. The two black circles mark the flexible joints or hinges Cβi-FGαi, which is relatively quiet. The two white circles point out the strong features caused by the ratcheted switches Cαi-FGβi. **c**. Same as **b** except expressed in percentage. **d**. Rmsd matrix. An rmsd matrix consists of rmsd values of all corresponding elements of many distance matrices of the same size. This rmsd matrix is calculated from distance matrices of 280 tetramers. αβ is quite rigid compared to quaternary changes across two dimers. The symmetry of inter-subunit squares observed in **b** also applies.
(TIFF)

Figure S3 **Decomposed lower triangles.** SVD analysis of 280 tetramers produces five significant decomposed lower triangles (Figure 2g). The first one is an average of all distance matrices of tetrameric Hb that resembles Figure S2a. Four other decomposed lower triangles with *k* = 2 - 5 are shown in **a** - **d**, respectively. Positive and negative values are indicated by blue and red colors as shown in the color bar on the right. Small values are shown in pale green. Column mean is plotted on the top of each panel. The second lower triangle (**a**) is very similar to the difference matrix calculated from the carbonmonoxy (2DN3) and deoxy (2DN2) structures (Figure S2b), since T-R difference is largely along the composition of the second component (Figures 2a and 2b). It is also very similar to the rmsd matrix (Figure S2d) except that the sign in the rmsd matrix is lost. That is to say, the second decomposed lower triangle captures the largest motions in the tetramer.
(TIFF)

Figure S4 **Saving in path length of traveling salesman problem.** Saving in total path length upon bypassing one tetrameric structure shows that 1YZI stands out from all others.
(TIFF)

Figure S5 **Hypothetical structures spanning the allosteric taboo gap.** All structures are aligned together by least-squares fitting of the invariant framework of the bottom dimer (Figure S11). Two proximal His residues move towards each other from T to R, while two distal His show little motion. Two β C-terminal His residues swing towards each other during T-R transition (outlined arrows). This motion continues until they contact each other in R2 (not shown). α and β are in pink and light blue, respectively. Some parts of the structure are removed for clarity.
(TIFF)

Figure S6 **The first four dimensions of α and β conformational spaces.** The coefficient sets *w*_*k*_ *V*_*k*_ with *k* = 1, …, 4 are derived from SVD analysis of 560 αs (**a** - **c**) and 560 βs (**d** - **f**). The continuous color scheme is the same as in Figure 2. In addition, all subunits on T side of the allosteric taboo gap are represented by open circles, and those on R side are in solid dots. Two counterpart subunits from a same tetramer are linked by a gray line. PDB entries are labeled by small typeface whenever possible, and are only visible on a digital copy. The decomposed lower triangles are in Figure S7.
(TIF)

Figure S7 **Decomposed lower triangles by SVD of subunit distance matrices.** See also the legends of Figures S3 and S6.
(TIFF)

Figure S8 **Decomposed lower triangles for combined conformational space of α and β.** See also the legends of Figures 3 and S3.
(TIFF)

Figure S9 **A small portion of all HIMs for SVD analysis.** HIMs are calculated from 560 dimers for SVD analysis. PDB entries are labeled on the left. Each label corresponds to two HIMs from the two αβ dimers. Helices are marked at the top and bottom. See Figure 5 legend for the color coding. A careful inspection at Fα may help to notice a transition from T state at the lower half of the figure to R state at the upper half. SVD analysis reveals the transition in great detail. This figure also opens a tiny window that displays the underlying data in the SVD analysis of distance matrices. Some noise is visible from lower resolution entries such as 1LFZ at 3.1 Å.
(TIFF)

Figure S10 **Allosteric core and distal block.** α and β are in warm and cool colors, respectively. The allosteric cores and distal blocks are in darker and lighter colors. **a**. Side view with dimer interface facing up. **b**. Top view directly into the dimer interface from the opposite dimer.
(TIFF)

Figure S11 **Rmsd matrices.** Larger rmsd values in darker blues indicate greater structural mobility. Small values in pale green indicate invariant structural segments. Black squares on the major diagonal outline the internally rigid structural segments automatically identified. Black rectangles off the major diagonal mark the inter-segment variation. All segments must exhibit both small internal variation and small inter-segment variation to be part of the invariant structural framework. That is to say, the submatrix outlined by the black squares and rectangles must have a small average value. An automated procedure evaluates the penalty upon expanding the submatrix and the saving gained by shrinking the submatrix [5]. **a**. αβ. **b**. α. **c**. β.
(TIFF)

Figure S12 **Comparison of the N-terminal section A-B.** **a**. α in T and R states are in light and dark pink. **b**. β in T and R states are in light blue and blue. **c**. β in T and R2 states are in light and dark blue. **d**. β in T and R states of goose Hb (1A4F) are in light blue and blue.
(TIFF)

Figure S13 **Interhelix B-E junctions.** **a**. α. **b**. β. Gly59αC^*α*^ is 3.5 Å from the peptide plane of Gly25α-Ala26α, so is Gly64βC^*α*^ from the peptide plane of Gly24β-Gly25β.
(TIFF)

Figure S14 **The first five dimensions of the conformational space of Hb dimer.** The coefficient sets *w*_*k*_ *V*_*k*_ with *k* = 1, …, 5 are derived from SVD analysis of 560 dimers. The continuous color scheme is the same as in Figure 2. In addition, all subunits on T side of the allosteric taboo gap are represented by open circles, and those on R side are in solid dots. Two dimers from a same tetramer are linked by a gray line. PDB entries are labeled by small typeface whenever possible, and are only visible on a digital copy.
(TIFF)

Figure S15 **Decomposed lower triangles of distance matrices of dimers.** See also the legends of Figures S3 and S14.
(TIFF)

Figure S16 **Joint SVD analysis with dimers in the abnormal homotetrameric Hbs.** The coefficient sets *w*_*k*_ *V*_*k*_ with *k* = 3, 4, and 5 are plotted. These scatter plots are equivalent to Figure S14b, c. The second dimension *k* = 2 is an extra dimension needed to describe major difference between αβ and β_2_ or γ_2_ dimers. PDB entries are labeled by small typeface whenever possible, and are only visible on a digital copy.
(TIFF)

Movie S1 **Difference distance matrices along the spline trajectory.** Distance matrices are recomposed along the spline trajectory (Figure 2) at equal spacing. The midpoint of the allosteric taboo gap is chosen as a reference point, and subtracted from all recomposed distance matrices. This movie shows the evolution of inter-atomic distance changes along the trajectory. Distance geometry is applied to this series and produces the smooth structural changes displayed in Movies S2 and S3. See also Figure S3 legend.
(GIF)

Movie S2 **Back calculated structures along the spline trajectory.** α and β are in pink and light blue, respectively. Three phases of the quaternary rotation is visible from this movie. See also Movie S1 legend.
(GIF)

Movie S3 **Back calculated structures along the spline trajectory.** Same as Movie S2 viewed from an orthogonal direction.
(GIF)

Movie S4 **Back calculated structures from composite distance matrices.** Viewed from the opposite dimer. See Figure 4a for detail.
(GIF)

Movie S5 **Back calculated structures from composite distance matrices.** Viewed from the opposite dimer. See Figure 4b for detail.
(GIF)
