# Supplementary figures and images for: Reaction Trajectory Revealed by a Joint Analysis of Protein Data Bank
Source: PLoS One. 2013 Nov 11;8(11):e77141. doi: 10.1371/journal.pone.0077141 (PMC3823880; doi:10.1371/journal.pone.0077141)

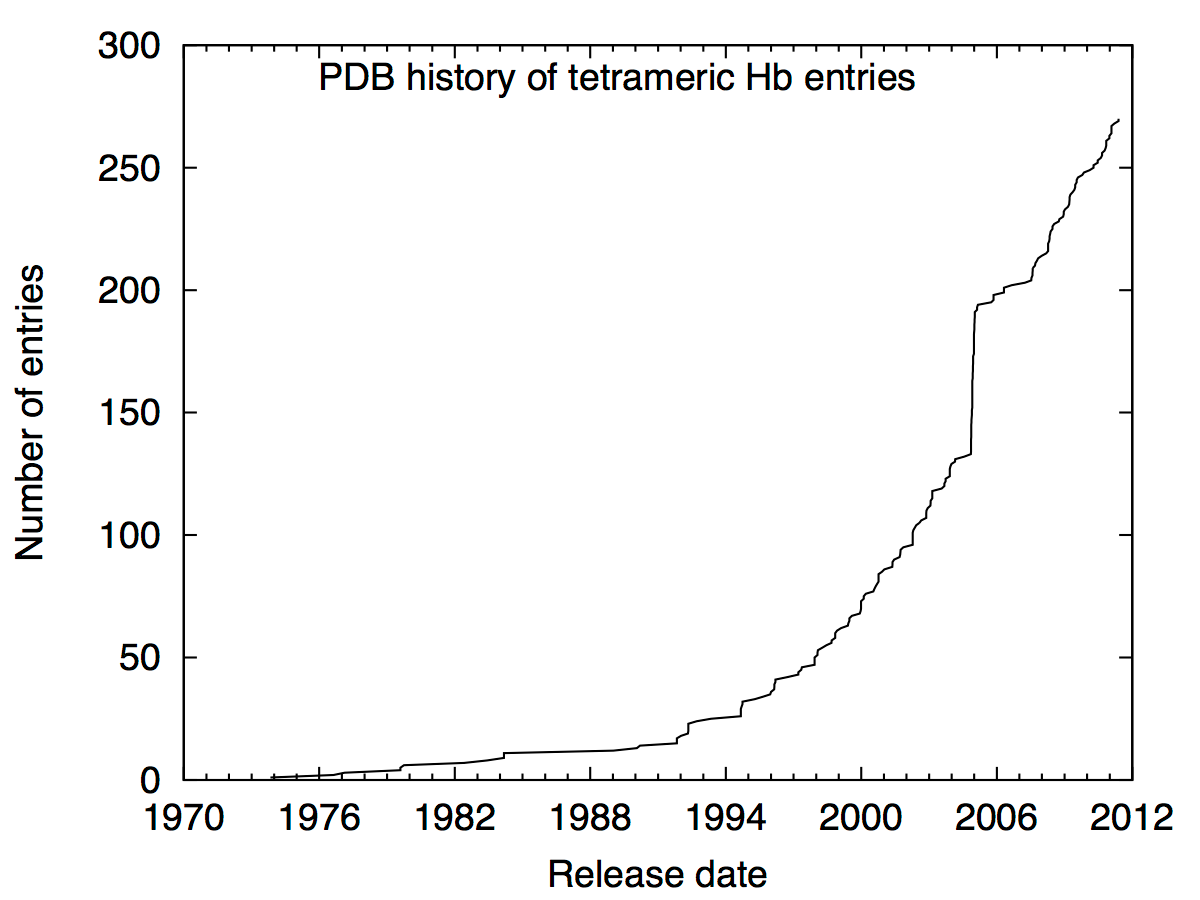

Supplement: Figure S1 — History of Hb entries. (TIFF) [file pone.0077141.s001.tiff]

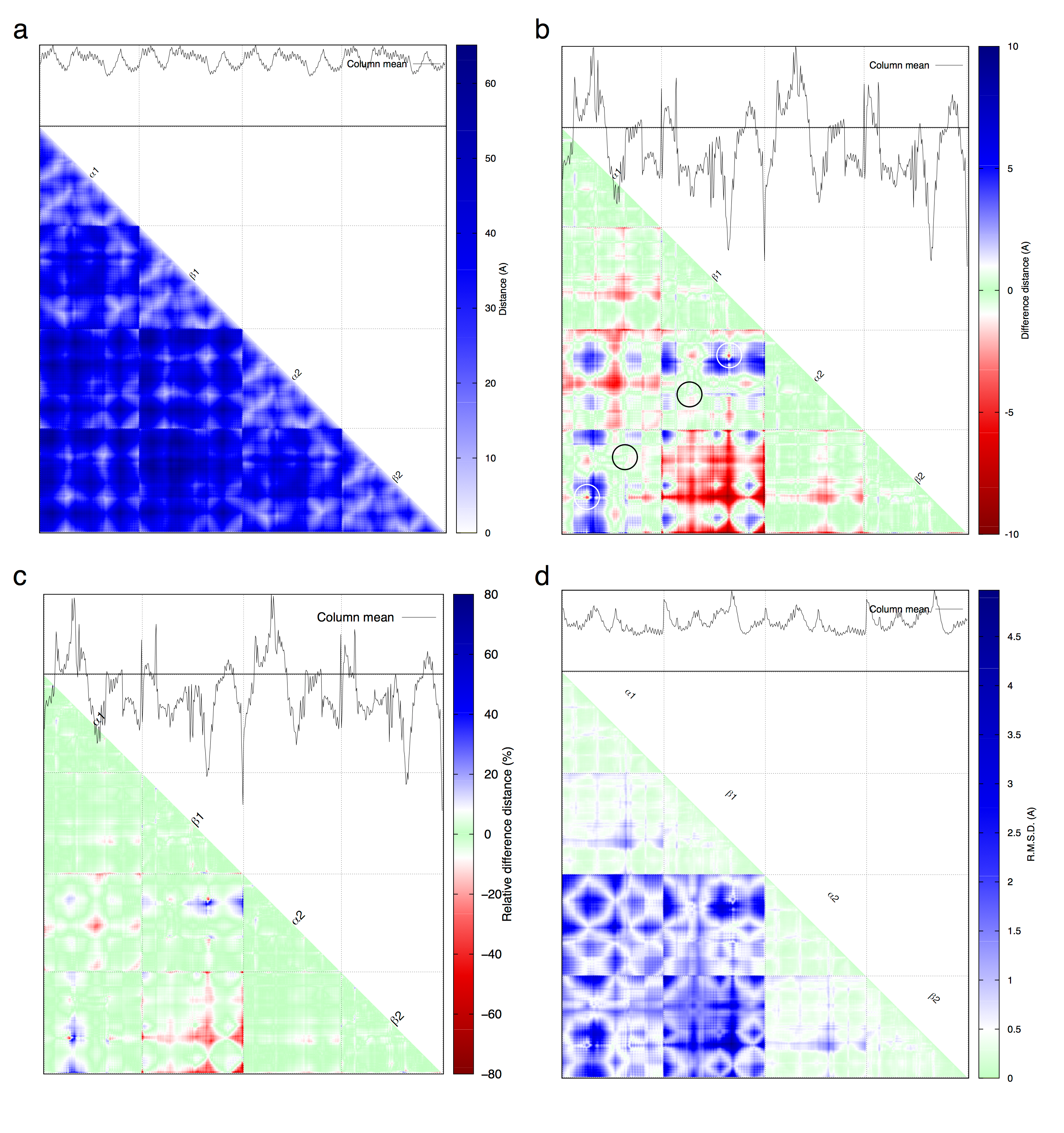

Supplement: Figure S2 — Distance matrix (a), difference matrix (b), relative difference matrix (c), and rmsd matrix (d). a. Distance matrix of the tetrameric deoxy Hb (2DN2). Four triangular portions of the matrix on the major diagonal contain intra-subunit distances. Each (nearly) square portion of the matrix contains inter-subunit distances. Column mean is plotted on top. b. Difference distance matrix. Difference is calculated between distance matrices of carbonmonoxy Hb (2DN3) and that in a. Positive and negative values are indicated by blue and red colors as shown in the color bar on the right. Small difference values are shown in pale green. The darkness of colors indicates that intra-dimer distance changes are greater than intra-subunit changes, but smaller than inter-dimer changes. All inter-subunit squares for partner and counterpart subunits are quite symmetrical about their major diagonals, such as α1-β1, α2-β2, α1-α2, and β1-β2. However, two inter-subunit squares for in-law subunits αi-βi are completely asymmetric. The symmetry of an inter-subunit square reflects the symmetry of the relative motion between these subunits. The two black circles mark the flexible joints or hinges Cβi-FGαi, which is relatively quiet. The two white circles point out the strong features caused by the ratcheted switches Cαi-FGβi. c. Same as b except expressed in percentage. d. Rmsd matrix. An rmsd matrix consists of rmsd values of all corresponding elements of many distance matrices of the same size. This rmsd matrix is calculated from distance matrices of 280 tetramers. αβ is quite rigid compared to quaternary changes across two dimers. The symmetry of inter-subunit squares observed in b also applies. (TIFF) [file pone.0077141.s002.tiff]

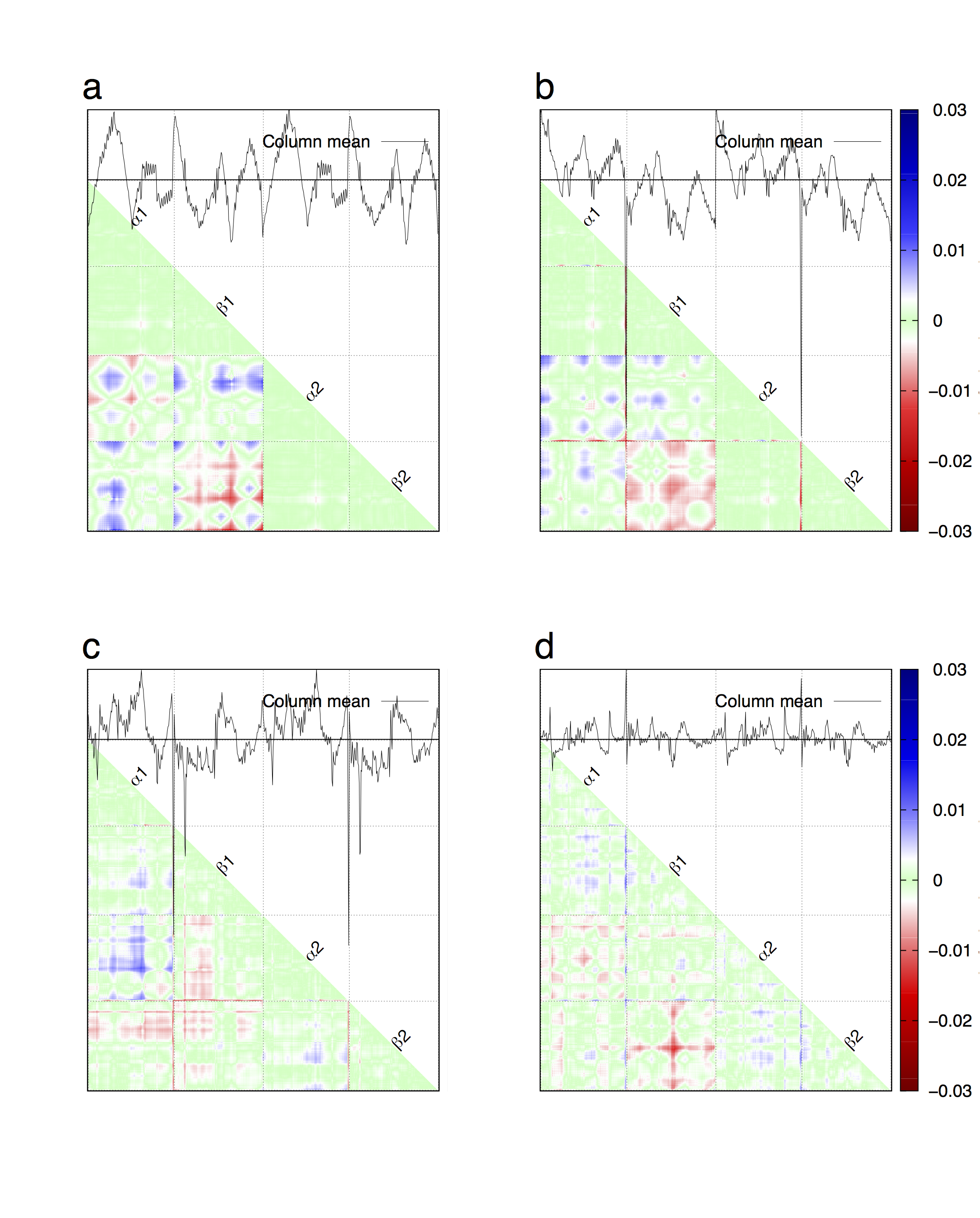

Supplement: Figure S3 — Decomposed lower triangles. SVD analysis of 280 tetramers produces five significant decomposed lower triangles (Figure 2g). The first one is an average of all distance matrices of tetrameric Hb that resembles Figure S2a. Four other decomposed lower triangles with k = 2 - 5 are shown in a - d, respectively. Positive and negative values are indicated by blue and red colors as shown in the color bar on the right. Small values are shown in pale green. Column mean is plotted on the top of each panel. The second lower triangle (a) is very similar to the difference matrix calculated from the carbonmonoxy (2DN3) and deoxy (2DN2) structures (Figure S2b), since T-R difference is largely along the composition of the second component (Figures 2a and 2b). It is also very similar to the rmsd matrix (Figure S2d) except that the sign in the rmsd matrix is lost. That is to say, the second decomposed lower triangle captures the largest motions in the tetramer. (TIFF) [file pone.0077141.s003.tiff]

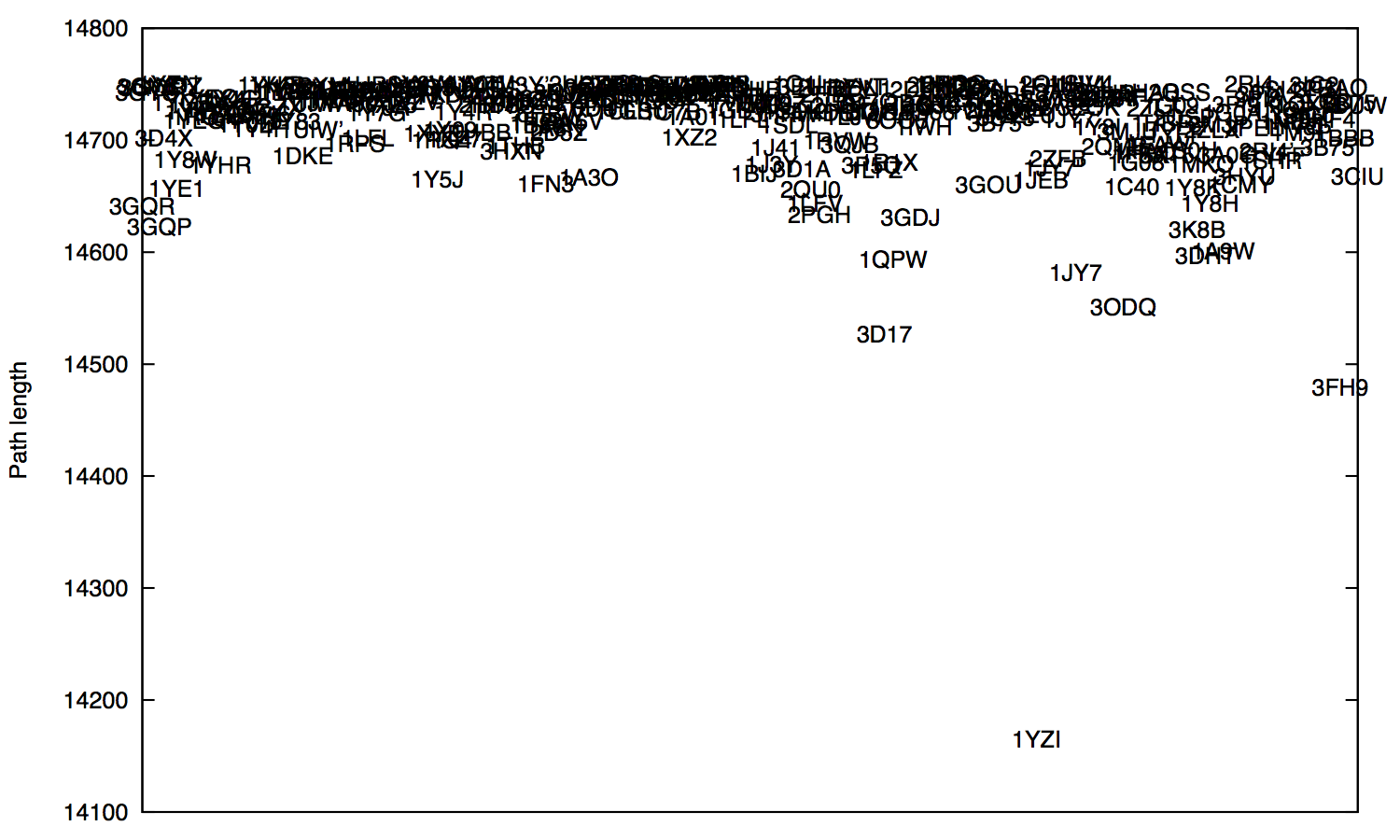

Supplement: Figure S4 — Saving in path length of traveling salesman problem. Saving in total path length upon bypassing one tetrameric structure shows that 1YZI stands out from all others. (TIFF) [file pone.0077141.s004.tiff]

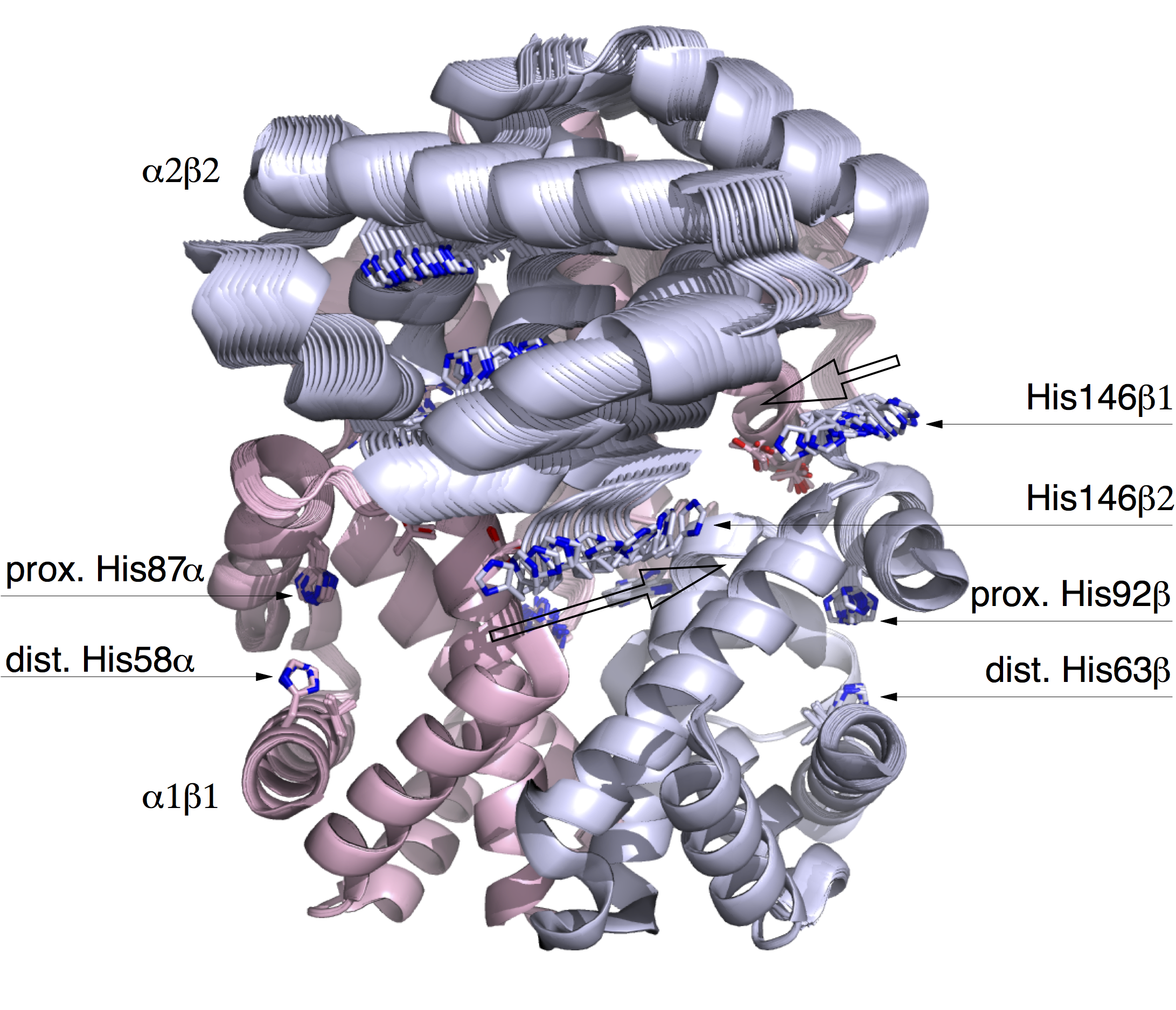

Supplement: Figure S5 — Hypothetical structures spanning the allosteric taboo gap. All structures are aligned together by least-squares fitting of the invariant framework of the bottom dimer (Figure S11). Two proximal His residues move towards each other from T to R, while two distal His show little motion. Two β C-terminal His residues swing towards each other during T-R transition (outlined arrows). This motion continues until they contact each other in R2 (not shown). α and β are in pink and light blue, respectively. Some parts of the structure are removed for clarity. (TIFF) [file pone.0077141.s005.tiff]

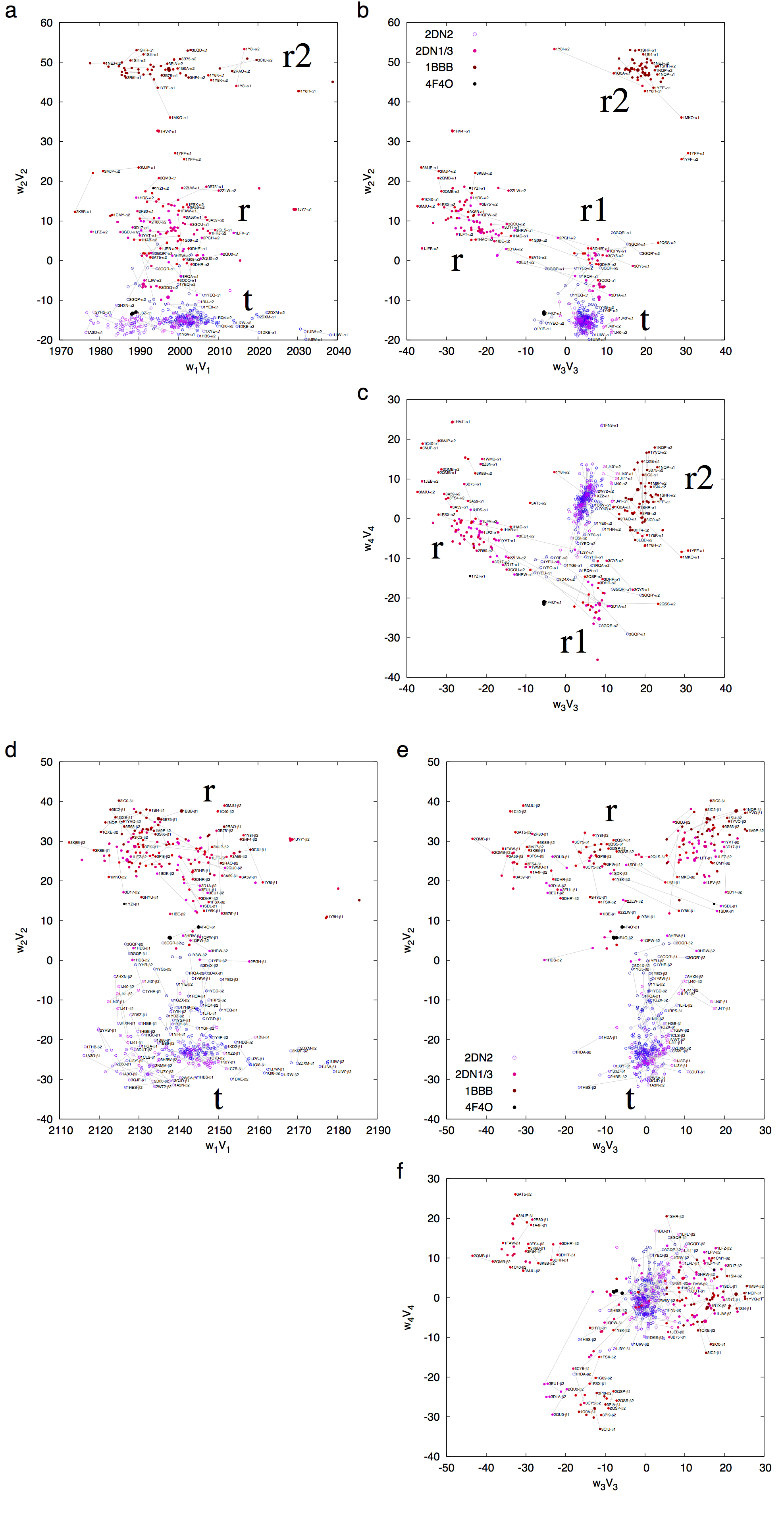

Supplement: Figure S6 — The first four dimensions of α and β conformational spaces. The coefficient sets w k V k with k = 1, …, 4 are derived from SVD analysis of 560 αs (a - c) and 560 βs (d - f). The continuous color scheme is the same as in Figure 2. In addition, all subunits on T side of the allosteric taboo gap are represented by open circles, and those on R side are in solid dots. Two counterpart subunits from a same tetramer are linked by a gray line. PDB entries are labeled by small typeface whenever possible, and are only visible on a digital copy. The decomposed lower triangles are in Figure S7. (TIF) [file pone.0077141.s006.tif]

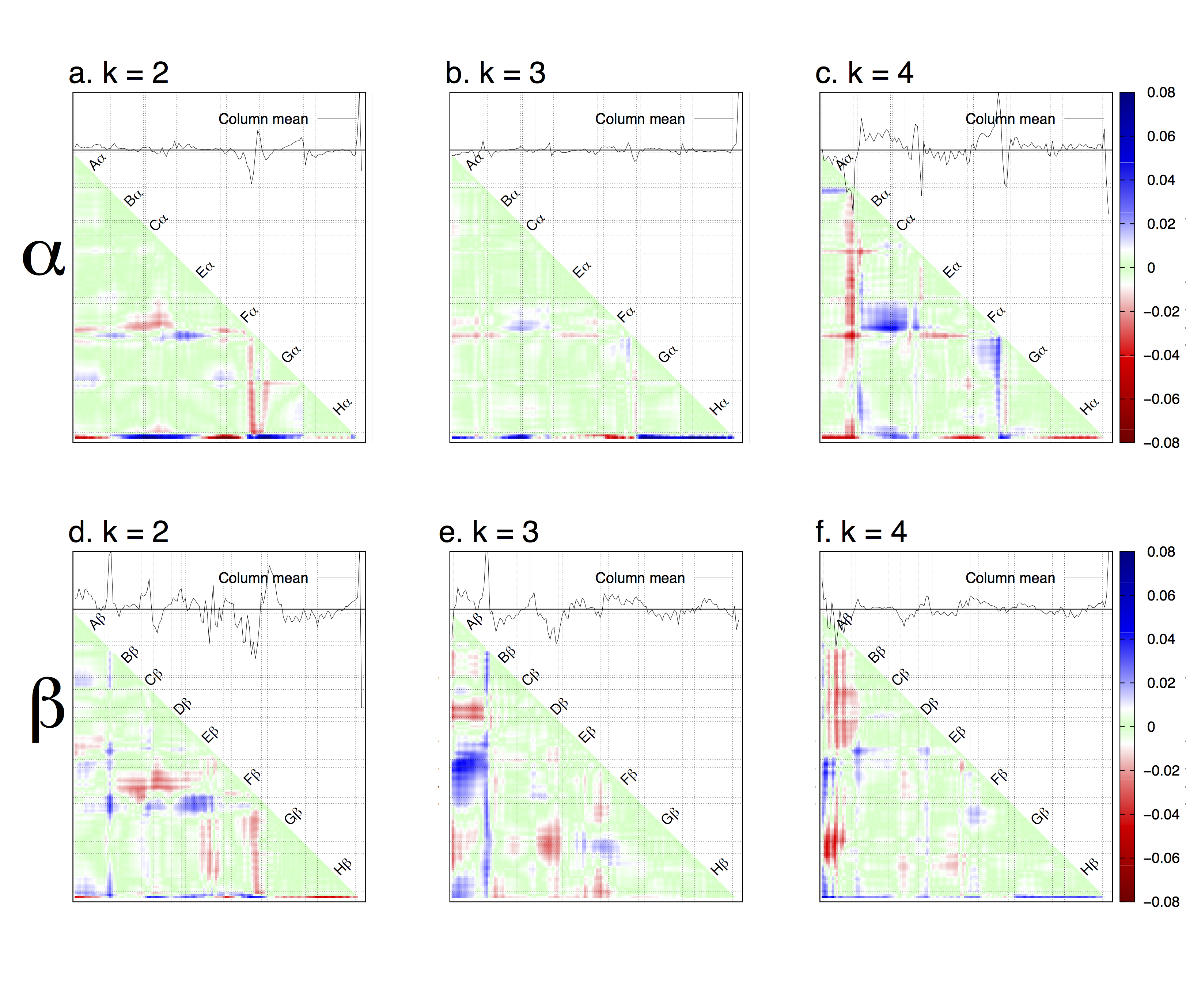

Supplement: Figure S7 — Decomposed lower triangles by SVD of subunit distance matrices. See also the legends of Figures S3 and S6. (TIFF) [file pone.0077141.s007.tiff]

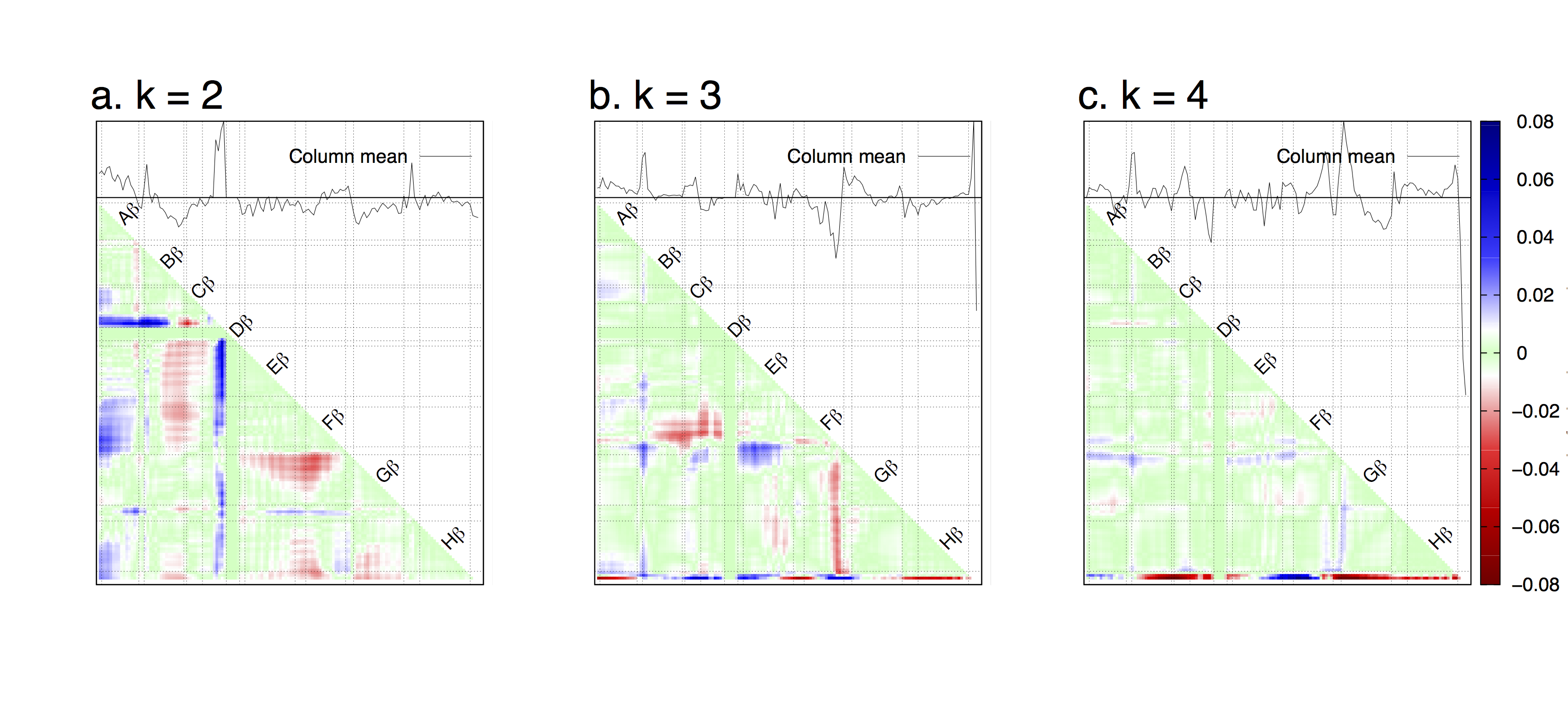

Supplement: Figure S8 — Decomposed lower triangles for combined conformational space of α and β. See also the legends of Figures 3 and S3. (TIFF) [file pone.0077141.s008.tiff]

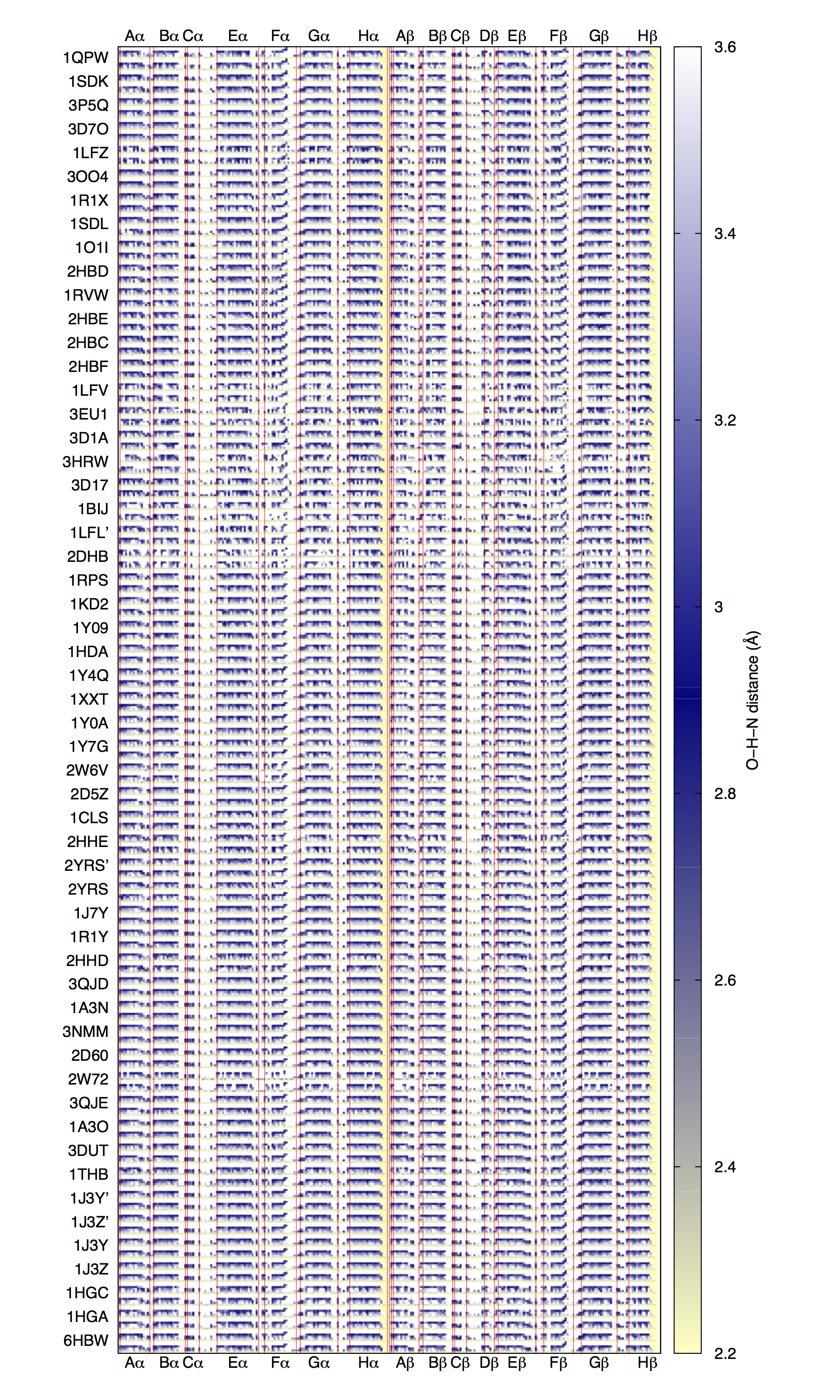

Supplement: Figure S9 — A small portion of all HIMs for SVD analysis. HIMs are calculated from 560 dimers for SVD analysis. PDB entries are labeled on the left. Each label corresponds to two HIMs from the two αβ dimers. Helices are marked at the top and bottom. See Figure 5 legend for the color coding. A careful inspection at Fα may help to notice a transition from T state at the lower half of the figure to R state at the upper half. SVD analysis reveals the transition in great detail. This figure also opens a tiny window that displays the underlying data in the SVD analysis of distance matrices. Some noise is visible from lower resolution entries such as 1LFZ at 3.1 Å. (TIFF) [file pone.0077141.s009.tiff]

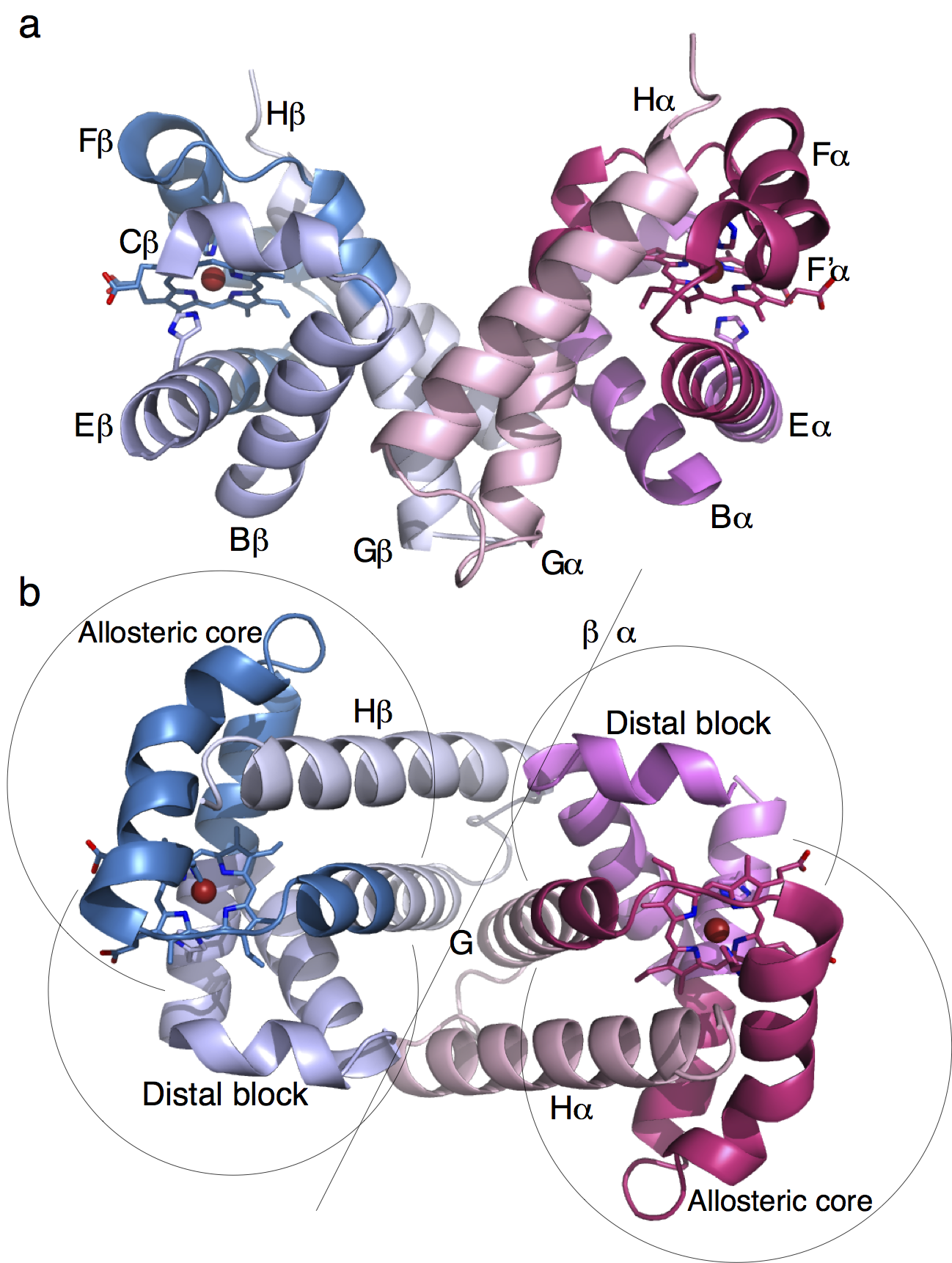

Supplement: Figure S10 — Allosteric core and distal block. α and β are in warm and cool colors, respectively. The allosteric cores and distal blocks are in darker and lighter colors. a. Side view with dimer interface facing up. b. Top view directly into the dimer interface from the opposite dimer. (TIFF) [file pone.0077141.s010.tiff]

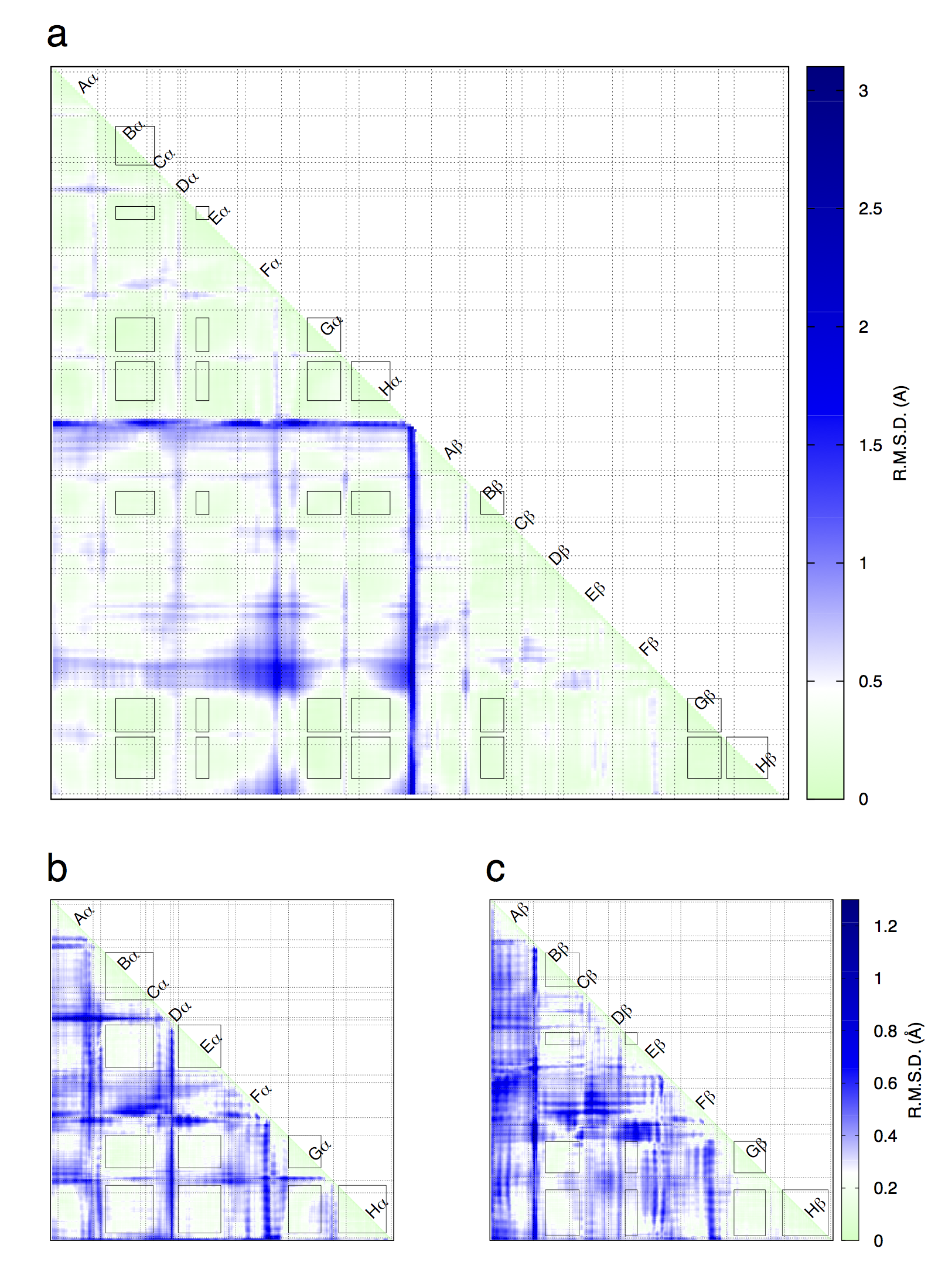

Supplement: Figure S11 — Rmsd matrices. Larger rmsd values in darker blues indicate greater structural mobility. Small values in pale green indicate invariant structural segments. Black squares on the major diagonal outline the internally rigid structural segments automatically identified. Black rectangles off the major diagonal mark the inter-segment variation. All segments must exhibit both small internal variation and small inter-segment variation to be part of the invariant structural framework. That is to say, the submatrix outlined by the black squares and rectangles must have a small average value. An automated procedure evaluates the penalty upon expanding the submatrix and the saving gained by shrinking the submatrix [5]. a. αβ. b. α. c. β. (TIFF) [file pone.0077141.s011.tiff]

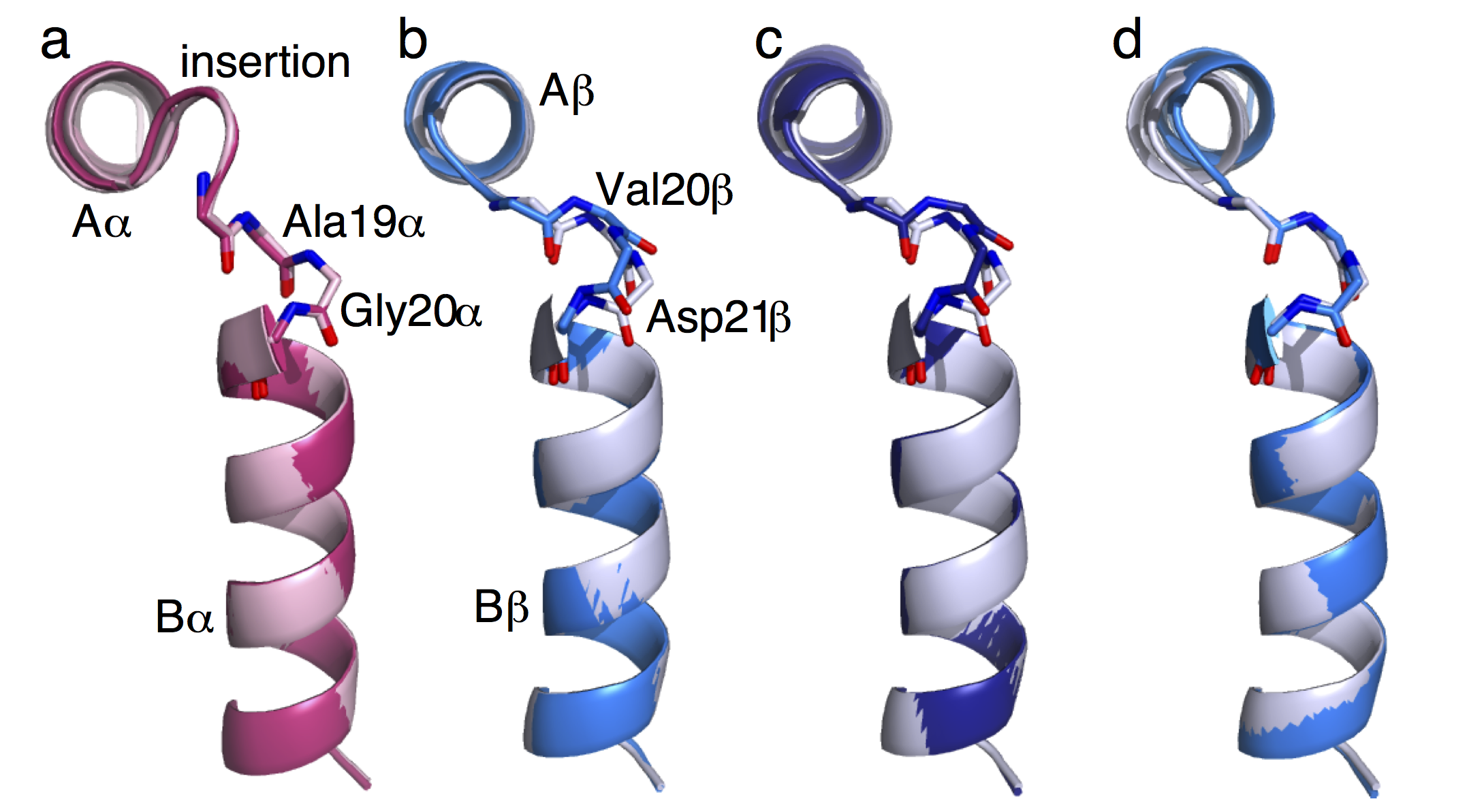

Supplement: Figure S12 — Comparison of the N-terminal section A-B. a. α in T and R states are in light and dark pink. b. β in T and R states are in light blue and blue. c. β in T and R2 states are in light and dark blue. d. β in T and R states of goose Hb (1A4F) are in light blue and blue. (TIFF) [file pone.0077141.s012.tiff]

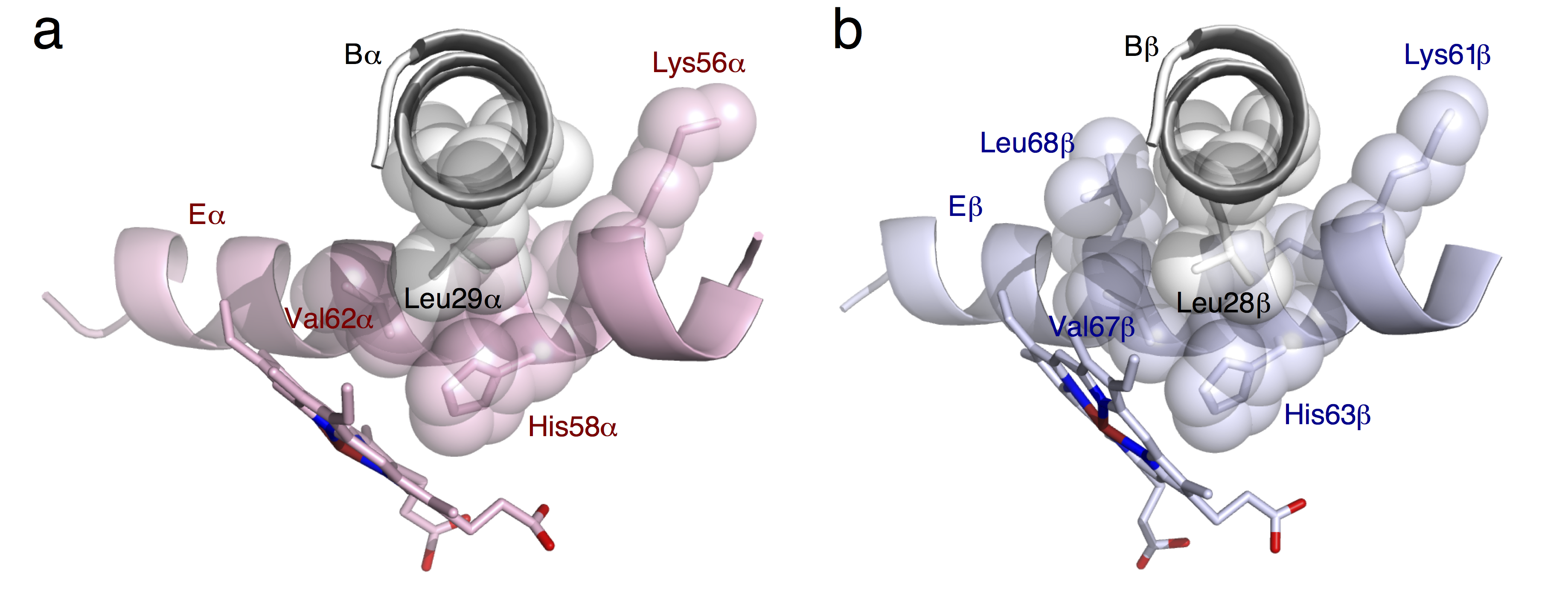

Supplement: Figure S13 — Interhelix B-E junctions. a. α. b. β. Gly59αCα is 3.5 Å from the peptide plane of Gly25α-Ala26α, so is Gly64βCα from the peptide plane of Gly24β-Gly25β. (TIFF) [file pone.0077141.s013.tiff]

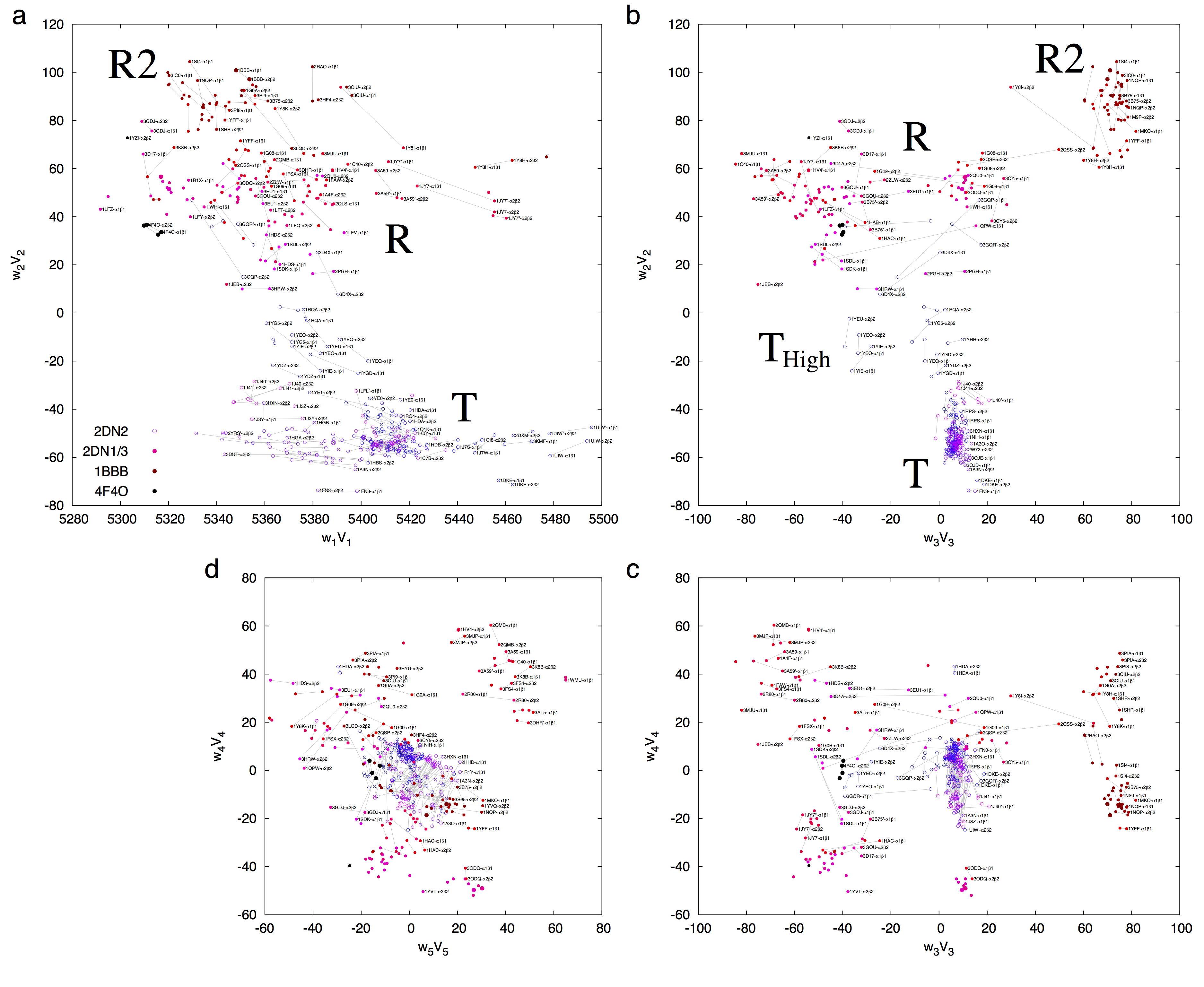

Supplement: Figure S14 — The first five dimensions of the conformational space of Hb dimer. The coefficient sets w k V k with k = 1, …, 5 are derived from SVD analysis of 560 dimers. The continuous color scheme is the same as in Figure 2. In addition, all subunits on T side of the allosteric taboo gap are represented by open circles, and those on R side are in solid dots. Two dimers from a same tetramer are linked by a gray line. PDB entries are labeled by small typeface whenever possible, and are only visible on a digital copy. (TIFF) [file pone.0077141.s014.tiff]

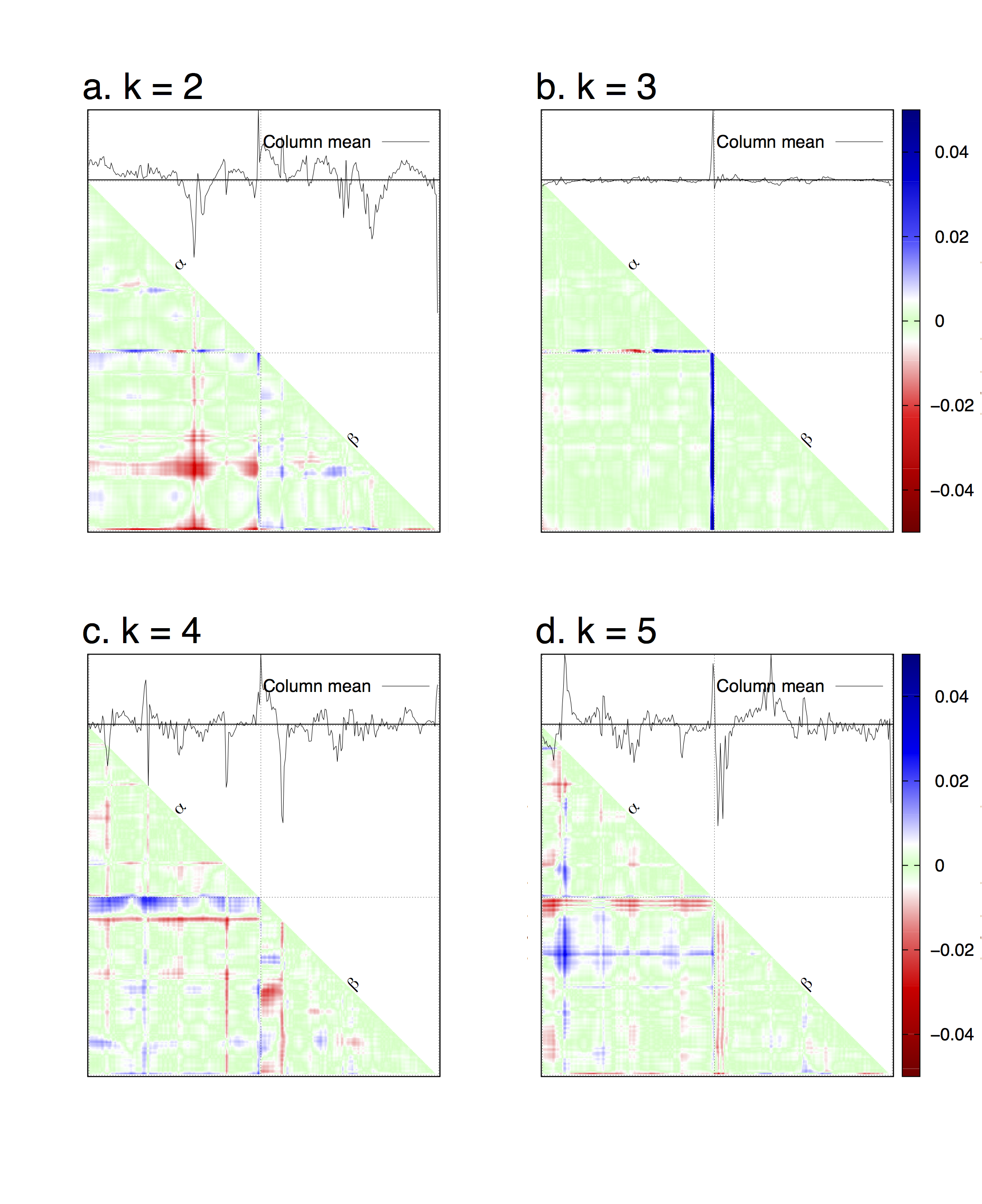

Supplement: Figure S15 — Decomposed lower triangles of distance matrices of dimers. See also the legends of Figures S3 and S14. (TIFF) [file pone.0077141.s015.tiff]

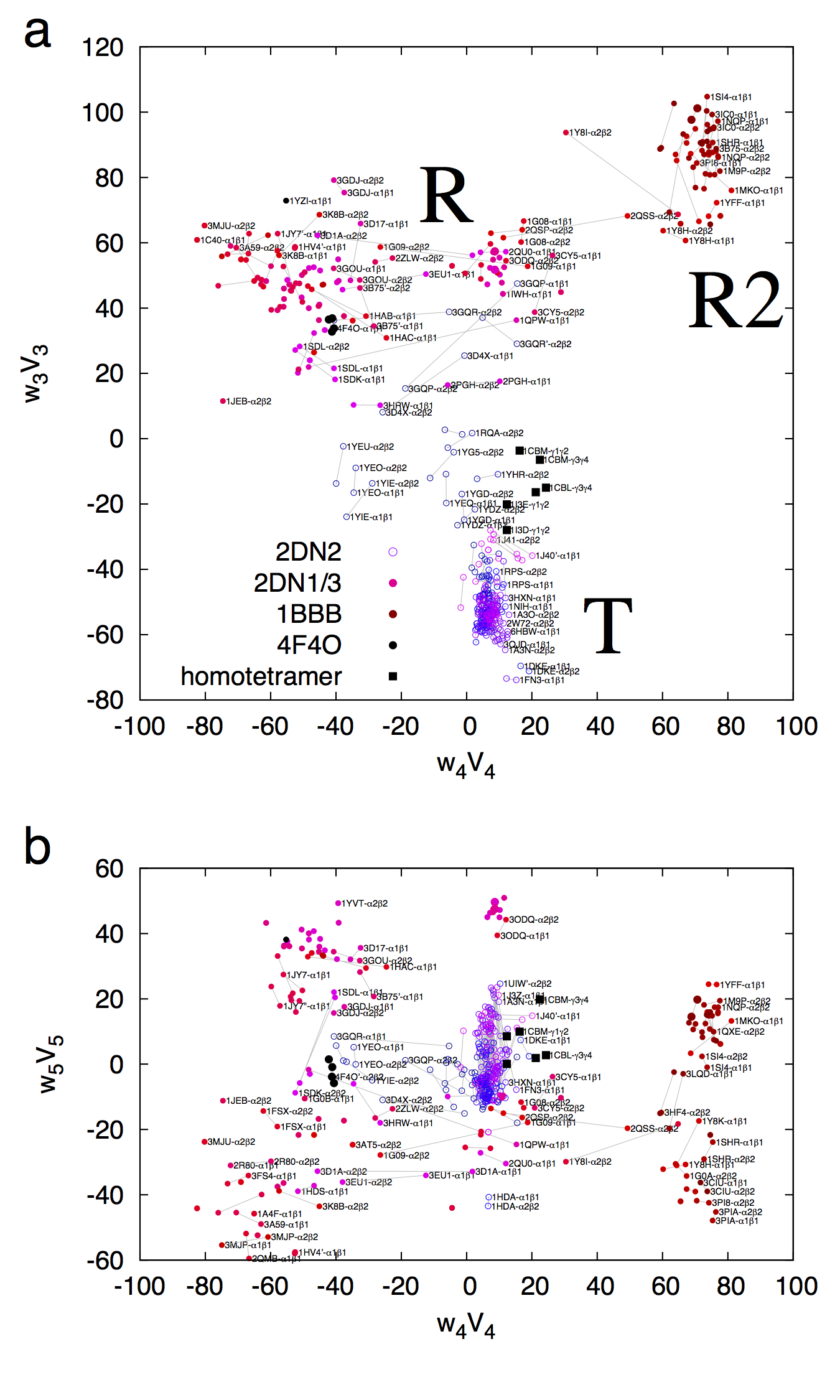

Supplement: Figure S16 — Joint SVD analysis with dimers in the abnormal homotetrameric Hbs. The coefficient sets w k V k with k = 3, 4, and 5 are plotted. These scatter plots are equivalent to Figure S14b, c. The second dimension k = 2 is an extra dimension needed to describe major difference between αβ and β2 or γ2 dimers. PDB entries are labeled by small typeface whenever possible, and are only visible on a digital copy. (TIFF) [file pone.0077141.s016.tiff]

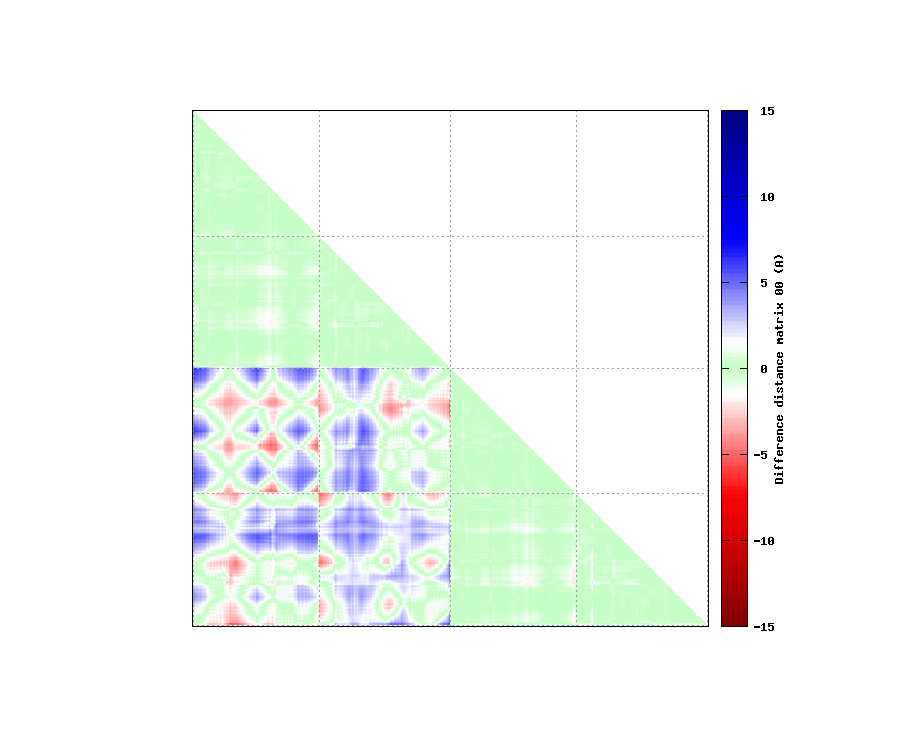

Supplement: Movie S1 — Difference distance matrices along the spline trajectory. Distance matrices are recomposed along the spline trajectory (Figure 2) at equal spacing. The midpoint of the allosteric taboo gap is chosen as a reference point, and subtracted from all recomposed distance matrices. This movie shows the evolution of inter-atomic distance changes along the trajectory. Distance geometry is applied to this series and produces the smooth structural changes displayed in Movies S2 and S3. See also Figure S3 legend. (GIF) [file pone.0077141.s017.gif]

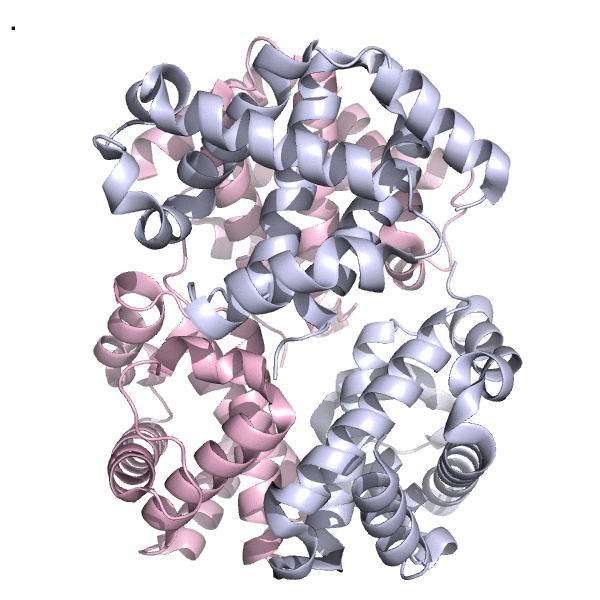

Supplement: Movie S2 — Back calculated structures along the spline trajectory. α and β are in pink and light blue, respectively. Three phases of the quaternary rotation is visible from this movie. See also Movie S1 legend. (GIF) [file pone.0077141.s018.gif]

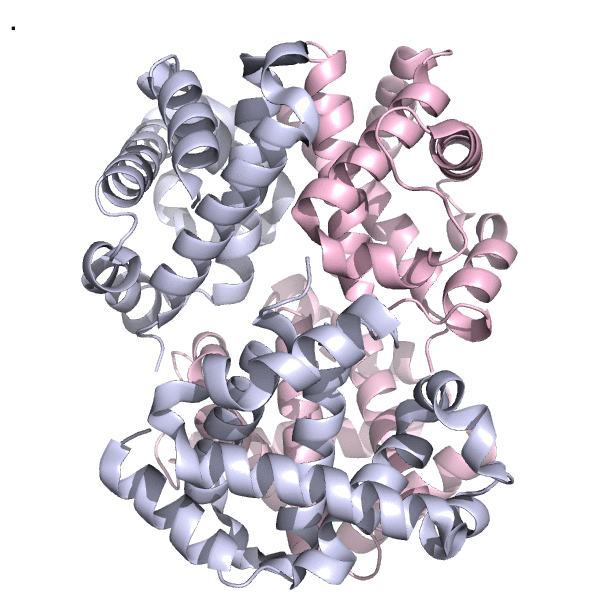

Supplement: Movie S3 — Back calculated structures along the spline trajectory. Same as Movie S2 viewed from an orthogonal direction. (GIF) [file pone.0077141.s019.gif]

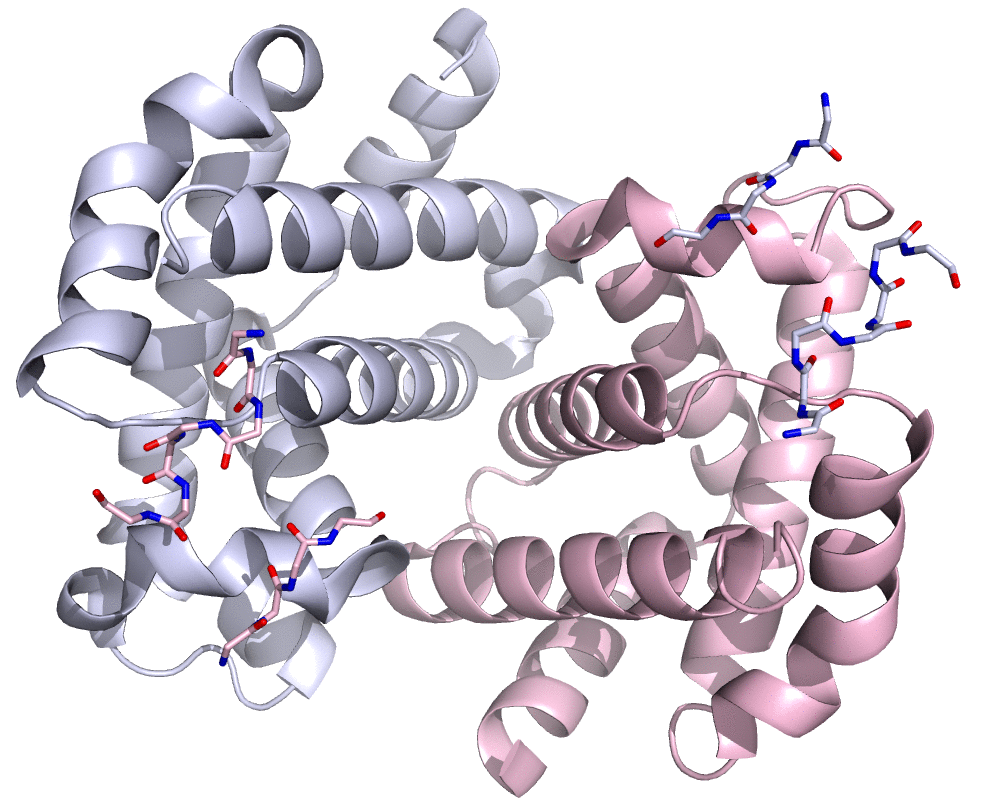

Supplement: Movie S4 — Back calculated structures from composite distance matrices. Viewed from the opposite dimer. See Figure 4a for detail. (GIF) [file pone.0077141.s020.gif]

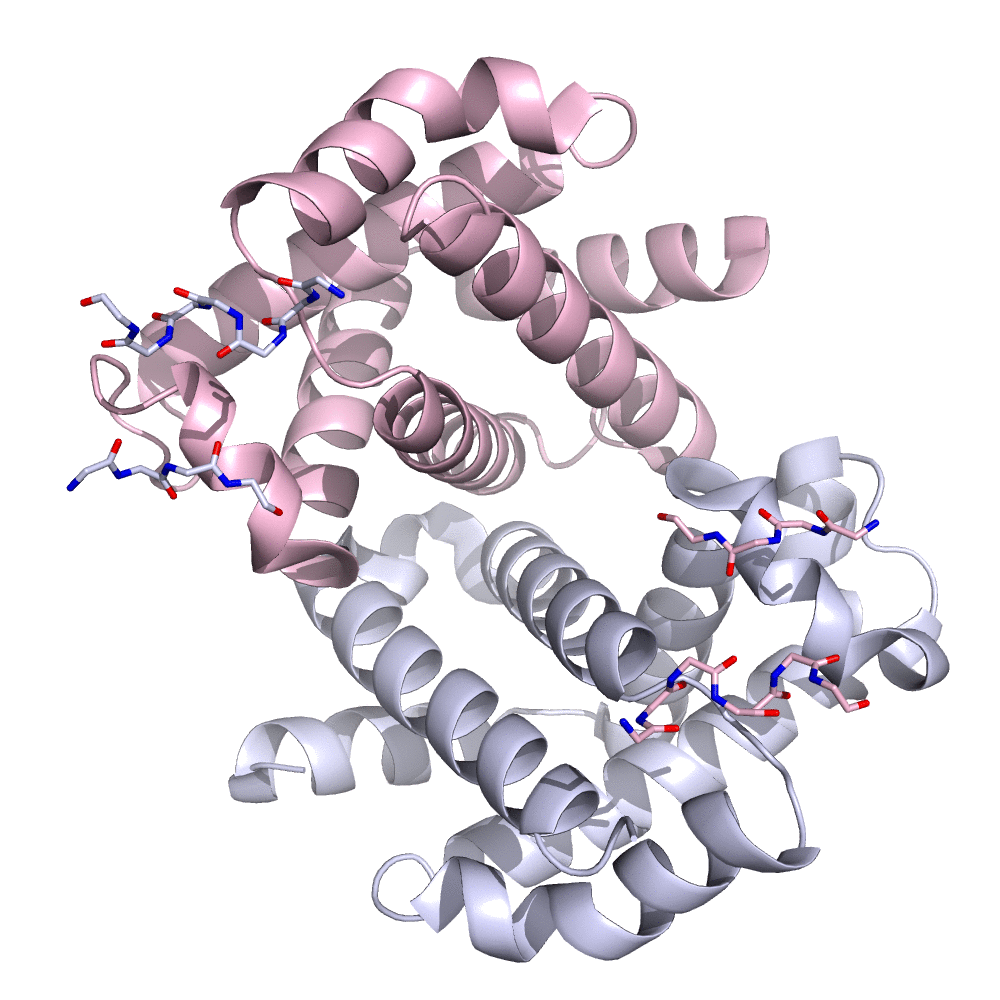

Supplement: Movie S5 — Back calculated structures from composite distance matrices. Viewed from the opposite dimer. See Figure 4b for detail. (GIF) [file pone.0077141.s021.gif]
